# Human intestinal epithelial cells can internalize luminal fungi *via* LC3-associated phagocytosis

**DOI:** 10.3389/fimmu.2023.1142492

**Published:** 2023-03-08

**Authors:** Sarit Cohen-Kedar, Efrat Shaham Barda, Keren Masha Rabinowitz, Danielle Keizer, Hanan Abu-Taha, Shoshana Schwartz, Kawsar Kaboub, Liran Baram, Eran Sadot, Ian White, Nir Wasserberg, Meirav Wolff-Bar, Adva Levy-Barda, Iris Dotan

**Affiliations:** ^1^ Division of Gastroenterology, Rabin Medical Center, Petah-Tikva, Israel; ^2^ Felsenstein Medical Research Center, Sackler Faculty of Medicine, Tel-Aviv University, Tel-Aviv, Israel; ^3^ Division of Surgery, Rabin Medical Center, Petah-Tikva, Israel; ^4^ Sackler Faculty of Medicine, Tel Aviv University, Tel Aviv, Israel; ^5^ Department of Pathology, Rabin Medical Center, Petah-Tikva, Israel; ^6^ Biobank, Department of Pathology, Petah-Tikva, Israel

**Keywords:** Candida albicans, intestinal epithelial cells, LC3-associated phagocytosis, organoids, dectin-1

## Abstract

**Background:**

Intestinal epithelial cells (IECs) are the first to encounter luminal microorganisms and actively participate in intestinal immunity. We reported that IECs express the β-glucan receptor Dectin-1, and respond to commensal fungi and β-glucans. In phagocytes, Dectin-1 mediates LC3-associated phagocytosis (LAP) utilizing autophagy components to process extracellular cargo. Dectin-1 can mediate phagocytosis of β-glucan-containing particles by non-phagocytic cells. We aimed to determine whether human IECs phagocytose β-glucan-containing fungal particles *via* LAP.

**Methods:**

Colonic (n=18) and ileal (n=4) organoids from individuals undergoing bowel resection were grown as monolayers. Fluorescent-dye conjugated zymosan (β-glucan particle), heat-killed- and UV inactivated *C. albicans* were applied to differentiated organoids and to human IEC lines. Confocal microscopy was used for live imaging and immuno-fluorescence. Quantification of phagocytosis was carried out with a fluorescence plate-reader.

**Results:**

zymosan and *C. albicans* particles were phagocytosed by monolayers of human colonic and ileal organoids and IEC lines. LAP was identified by LC3 and Rubicon recruitment to phagosomes and lysosomal processing of internalized particles was demonstrated by co-localization with lysosomal dyes and LAMP2. Phagocytosis was significantly diminished by blockade of Dectin-1, actin polymerization and NAPDH oxidases.

**Conclusions:**

Our results show that human IECs sense luminal fungal particles and internalize them *via* LAP. This novel mechanism of luminal sampling suggests that IECs may contribute to the maintenance of mucosal tolerance towards commensal fungi.

## Introduction

Intestinal epithelial cells (IECs) stand in the frontline of the largest mucosal surface in the human body. As such, IECs are in constant interaction with luminal microorganisms and dietary molecules, as well as with immune cells in the lamina propria beneath them. Besides being a physical barrier, IECs function as innate immune cells, sensing and actively responding to luminal microbiota (1–3). However, the role of human IECs in host tolerance towards commensal fungi and their contribution to shaping host immune response remain obscure.

We previously reported that human IECs express the β-glucan receptor Dectin-1 (4), a central C-type-lectin-receptor (CLR) involved in fungal recognition and immune response (5–9). We further demonstrated that IECs were directly activated by cell wall components of commensal fungi *via* Dectin-1. However, pro-inflammatory cytokine secretion occurring in response to β-glucan was silenced when whole fungi were sensed (10) suggesting epithelial tolerance to commensals.

We therefore sought a physiological homeostatic outcome of fungal recognition by IECs. Dectin-1 functions in phagocytosis of non-opsonized fungi by professional phagocytes (9, 11, 12). As stable transfection of Dectin-1 allows β-glucan-dependent phagocytosis by non-phagocytes (11, 13, 14), we asked whether IECs, that endogenously express Dectin-1, can phagocytose β -glucan containing fungal particles, and by which mechanism.

Here we provide evidence supporting a novel mechanism of interaction between IECs and commensal fungi at the intestinal mucosa, where β-glucan and *C. albicans* were phagocytosed by human IECs in a Dectin-1 dependent and spleen tyrosine kinase (Syk) independent manner leading to LC3 associated phagocytosis (LAP) and lysosomal degradation.

## Results

### Zymosan uptake by IECs is dependent on actin-polymerization

**Figure 1:**
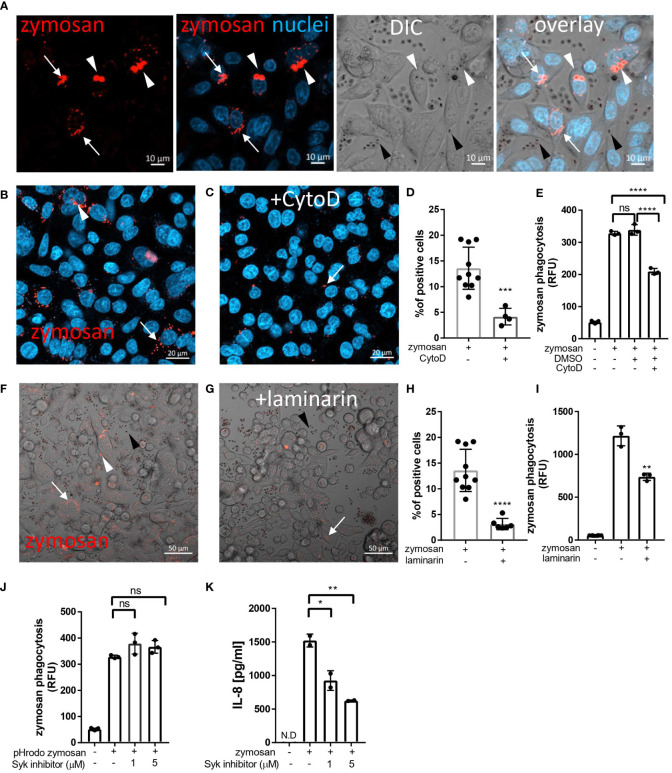
Uptake of zymosan by human intestinal epithelial cells. **(A)** SW480 cells were seeded on glass-bottom chambers as indicated in Methods, and fed overnight with pHrodo-red zymosan (zymosan, red) and counter stained with Hoechst 33342 (blue) prior to confocal live imaging. White arrowheads – intracellular red fluorescent zymosan, Black arrowheads - extracellular intact zymosan, arrow- intracellular fragmented zymosan. Original magnification x20, scale bar 10 µm. **(B–E)** Zymosan uptake is sensitive to cytochalasin-D. **(B, C)** SW480 were treated as in A, in the absence **(B)** or presence **(C)** of cytochalasin-D (CytoD, 10 µM). Scale bar 20 µm. Arrows and arrowhead indicate intracellular processed and intact zymosan respectively. Wider fields of the images are shown is **Supplementary Figure S3**. **(D)** Phagocytosis was quantified using imageJ as the percentage of red-fluorescence positive cells in at least 4 randomly taken fields as described in Methods. Each dot is the quantification of a single field. Data is representative of three independent experiments performed. ***p ≤ 0.001, Unpaired t-test vs. no inhibitor. **(E)** SW480 cells were seeded in 96 well plate, treated as in **(B, C)** as well as with the vehicle (DMSO, 1:1000) in triplicate wells, and phagocytosis was assessed as the relative fluorescence (RFU) by a microplate reader. Data are shown as the individual measure of each biological replica and mean ± SD of biological triplicates from a representative of three independent experiments performed. ns-non significant ****p<0.0001, One-way ANOVA followed by Tukey multiple comparison test. **(F–I)** Zymosan uptake depends on Dectin-1. **(F, G)** SW480 were treated as in A, in the absence **(F)** or presence **(G)** of laminarin (1 mg/ml) that was added to the medium 1 hour prior to zymosan. Scale bar 50 µm. White arrows and arrowhead indicate intracellular processed and intact zymosan respectively. Black arrowheads indicate extracellular zymosan. **(H)** Phagocytosis was quantified as in **(D)**. **(I)** cells were seeded on 96 wells, treated as in **(F, G)** in triplicate wells, and phagocytosis was analyzed as in **(E)**. **(J)** Zymosan phagocytosis is resistant to Syk inhibition. SW480 cells were seeded on 96 well plate, in the presence or absence of the Syk inhibitor 574711 (1 and 5 µM), which was added 1 hour prior to the addition of pHrodo-red zymosan. Phagocytosis was assessed as in **(E)**. Data are shown as individual measures and mean ± SD of biological triplicates from a representative of three independent experiments performed. **(K)** Zymosan-induced IL-8 secretion is sensitive to Syk inhibitor. Cells seeded on the same 96 well plate were pre-treated with Syk inhibitor as in **(J)** and stimulated overnight with 100 μg/ml of non-labelled zymosan. Supernatants were assessed for IL-8 by ELISA. Data are shown as individual measures and mean ± SD of biological duplicates from a representative of three independent experiments performed. N.D- not detected; ns-non significant *p<0.05; **p<0.01, One-way ANOVA followed by Tukey multiple comparison test.

To assess phagocytosis in IECs we chose zymosan, a particulate β-glucan rich, cell wall extract of *Saccharomyces cerevisiae* which is commensal in the human gut. Hence, zymosan is highly applicable as a representative of luminal fungal species in the vicinity of mucosal surfaces. In addition, zymosan is widely used to study phagocytosis by professional phagocytes and the pHrodo-red label of zymosan, that turns fluorescent intracellularly, is indicative of internalized particles in living cells (15). Using the human epithelial cell lines SW480, HCT116 and Caco-2 we detected cellular uptake of pHrodo-red zymosan where some cells presented multiple internalized particles as well as fragmented zymosan indicative of intracellular processing (**Figure 1A** and **Supplementary Figures S1A, S2A** respectively), suggesting that phagocytosis of β-glucan expressing particles is common in human IECs. We detected cellular internalization of pHrodo-red zymosan in a few cells as early as 3 hours of incubation and phagocytosis was clearly observed following overnight incubation where up to 20% of the cells were zymosan positive (**Figures 1B, D**).

To test whether cytoskeleton-mediated engulfment participates in the uptake of zymosan, we applied the actin- polymerization-inhibitor cytochalasin-D. This resulted in attenuated phagocytosis as reflected by a 70% decrease in the number of pHrodo-red zymosan positive SW480 cells (***p=≤0.001, **Figures 1B–D** and **Supplementary Figure S3**), and in the total fluorescence of intracellular pHrodo-red zymosan (**p ≤ 0.01, **Figure 1E**). Similar sensitivity to cytochalasin-D was observed in HCT116 cells (**Supplementary Figures S1B, C**). As actin mediated engulfment of extracellular particles is a hallmark of phagocytosis these results indicate a genuine zymosan phagocytosis in IECs.

### Zymosan phagocytosis by IECs involves Dectin-1

We have previously demonstrated functional Dectin-1 in IECs (4, 10). Since Dectin-1 mediates phagocytosis in professional phagocytes, we next asked whether it also functions in phagocytosis in IECs. To this end, we used laminarin, a soluble Dectin-1 antagonist, that blocks zymosan and fungal phagocytosis in professional phagocytes (16–19). Notably, laminarin inhibited zymosan phagocytosis by SW480 and HCT116 cells as indicated by significant decrease in the number of pHrodo-red zymosan positive cells (by 77% ****p ≤ 0.0001, **Figures 1F–H** and **Supplementary Figure S1B**) and the total fluorescence of the intracellular pHrodo-red zymosan (**Figure 1I** **p=≤0.01 and **Supplementary Figure S1D** ***p=≤0.001). Altogether Dectin-1 dependent zymosan phagocytosis by IEC lines was demonstrated.

### Phagocytosis by IECs is Syk-independent

Syk is a major signaling mediator downstream Dectin-1 and is involved in Dectin-1 triggered phagocytosis by professional phagocytes (19, 20). Yet, there are examples where Syk was dispensable for phagocytosis (13, 21). Syk is activated by commensal fungi and β-glucan and is required for β-glucan-induced cytokine secretion in human IECs (4, 10, 22). Therefore, we asked whether Syk is essential for zymosan phagocytosis. We found that the Syk inhibitor 574711 [3-(1-methyl-1H-indol-3-yl-methylene)-2-oxo-2,3-dihydro-1H-indole-5-sulfonamide] did not interfere with phagocytosis (**Figure 1J**) while its inhibitory activity was indicated by a significant decrease of zymosan-induced IL-8 secretion in the same experiment (60% inhibition by 5 µM of Syk inhibitor, **P ≤ 0.01, **Figure 1K**). Further evidence for Syk-independence was obtained with Caco-2 cells, which do not express Syk (10), but readily phagocytose pHrodo-red zymosan (**Supplementary Figure S2**). We conclude that in IECs, phagocytosis of zymosan may occur independently of Syk activation.

### Human intestinal organoids can phagocytose zymosan

**Figure 2:**
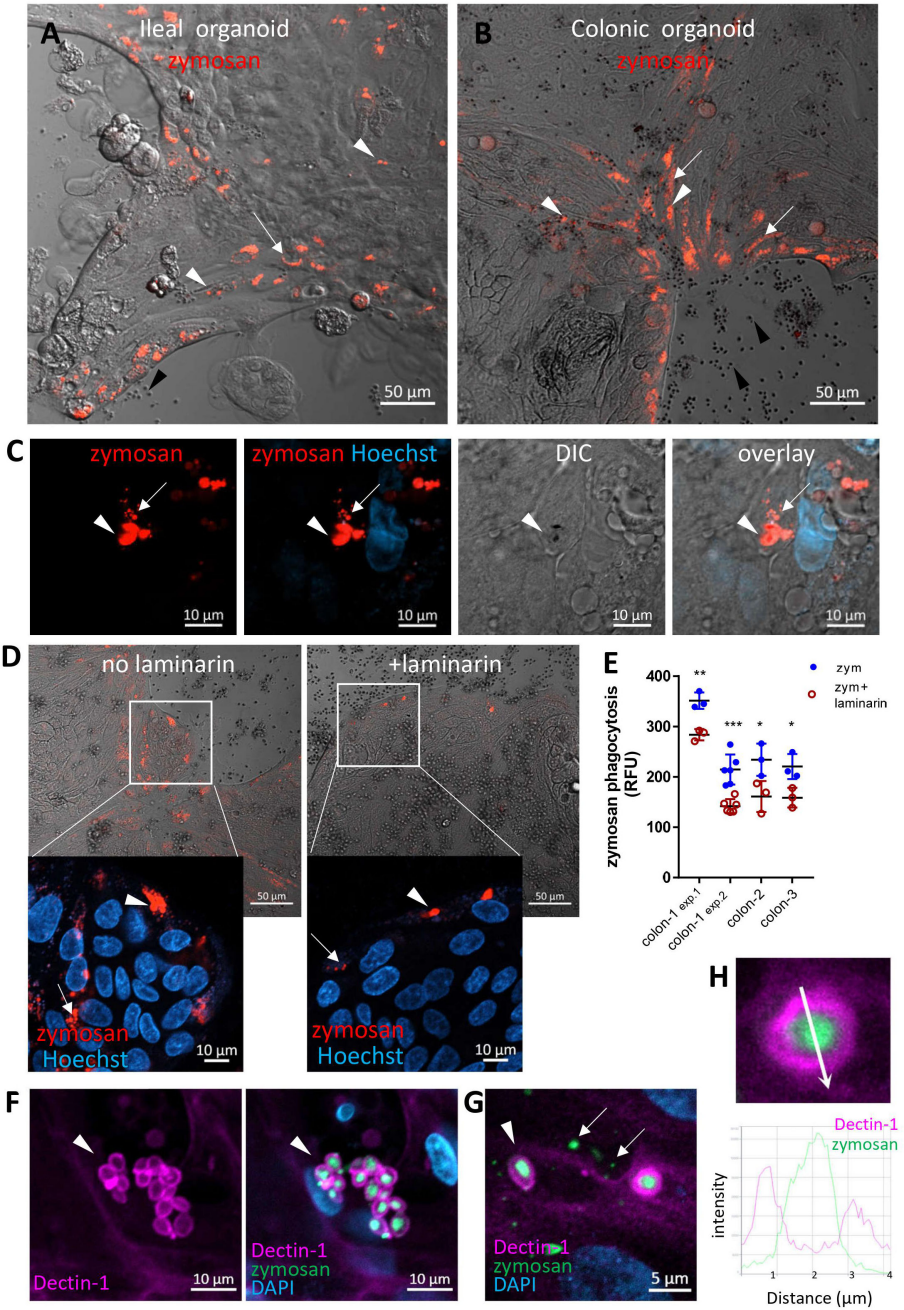
Intestinal organoids uptake zymosan. **(A, B)** Ileal **(A)** and colonic **(B)** organoids were grown as monolayers in expansion medium and let to differentiate for 2 days. pHrodo-red zymosan (red) was added to the medium for 24h. Original magnification x10, scale bar 50 µm, white arrows and arrowhead indicate intracellular processed and intact zymosan respectively. Black arrowheads indicate extracellular zymosan. Shown are representative frames from wider fields, presented in **Supplementary Figures S4, S5A**, from 3-5 randomly acquired scans of two independent experiments of two organoids. **(C)** Colonic organoids (from a different individual) were treated as in **(B)**, nuclei were stained with Hoechst 33342 (blue) prior to confocal live imaging. Shown a representative frame from a z-stack analysis. Entire z-stack movie is shown in **Supplementary Movie 1**. Arrows and arrowhead indicate intracellular processed and intact zymosan respectively. Original magnification x63, scale bar 10 µm. **(D)** Laminarin inhibits zymosan uptake by intestinal organoids. Colonic organoids were grown as in B, in the presence or absence of laminarin (1 mg/ml) that was added to the medium 1 hour prior to zymosan in triplicate wells. Shown are intracellular fluorescent zymosan (red) and the organoid cells (DIC), or zoom-in insets where nuclei were stained with Hoechst 33342 (blue) prior to confocal live imaging. Shown are representative frames from 3-6 random fields imaged from each of triplicate wells. The experiment was repeated with organoids from four individuals. Original magnification x20, scale bar 50 and 10 µm, Arrows and arrowhead indicate intracellular processed and intact zymosan respectively. **(E)** Colonic organoids from three individuals (colon-1 to colon-3) were seeded in 96 well plate, treated as in **(D)** in triplicate or 6-replicate wells for 48 hours. Phagocytosis was assessed as the relative fluorescence by a microplate reader. Data are shown as the measured value (dots) and mean ± SD of biological replicates from four independent experiments performed. Colon-1 was tested twice (exp.1 and 2), zym=zymosan. ***p<0.001, **p<0.01, *p<0.05 vs. no inhibitor, Student’s t-test was performed individually for each independent experiment. **(F, G)** Dectin-1 is recruited to internalized zymosan. Ileal organoids were fed with AF488-zymosan (green) overnight and stained with Dectin-1 antibody (magenta) and DAPI. Original magnification x20 scale bar 10 µm **(F)** and x63 scale bar 5 µm **(G)**. Arrowheads – intact zymosan, arrows- fragmented zymosan. **(H)** Fluorescence intensity profile along the arrow of an inset from **(G)** is shown on the graph.

We next asked whether primary human IECs can phagocytose zymosan. To test this, we used human intestinal organoids generated from ileal and colonic crypts obtained from surgical samples, that were grown as two-dimensional monolayers to facilitate epithelial exposure to large particles (23) (see methods). We assessed phagocytosis in ileal and colonic organoids cultured in expansion medium and then grown for 2-3 additional days in a generic differentiation medium (see methods) prior to pHrodo-red zymosan exposure (**Figures 2A, B**; **Supplementary Figures S4, S5A**). We found pronounced phagocytic activity, which was distinctly higher in organoids grown in differentiation medium compared to those cultured in expansion medium only (**Supplementary Figure S5B**). Hence, we performed our phagocytosis experiments in differentiation medium throughout this work. Phagocytosis of zymosan was observed by all ileal (n=4) and colonic (n=18, derived from ascending [n=13], transverse [n=2] or from sigmoid colon [n=3]) organoids tested, suggesting that phagocytic capacity of the epithelium is found along the lower human gastrointestinal tract. Importantly, 2 of the ileal and 5 of the colonic organoids were derived from patients with Crohn’s disease, and one colonic organoid from ulcerative colitis. All these IBD-derived organoids phagocytosed zymosan and behaved identically to those generated from normal tissue, by all the parameters examined throughout this report, yet, all the data presented hereby, are from normal organoids. Fragmentation of internalized (fluorescent) zymosan suggests its intracellular processing in organoid cells (**Figure 2C** and **Supplementary Movies 1, 2**), similarly to our observation in cell lines.

Notably, different epithelial cell types in the organoid monolayers were identified (**Supplementary Figures S6, S7**) and phagocytosis was observed in goblet as (MUC2^+^) as well as non-goblet (MUC2^-^) IECs (**Supplementary Figure S8**), suggesting that phagocytosis may be shared by different types of IECs. This appeared to be more prominent at the periphery of the organoid cultures, which represent the luminal regions of the villus or crypt (24). Interestingly, Dectin-1 was expressed at the peripheral regions of the organoids (**Supplementary Figure S9**) and at the apical side of the lumen-facing IECs in colonic crypts of frozen sections (**Supplementary Figure S10**). To test the function of Dectin-1 in phagocytosis, we assessed intracellular pHrodo-red zymosan in organoids in the presence or absence of laminarin. A decrease in intracellular pHrodo-red zymosan in the presence of laminarin (**Figure 2D**) was observed by live confocal microscopy. This observation was quantitatively confirmed by a significant decrease (*p ≤ 0.05 to ***p ≤ 0.001 in organoids from three individuals) in the fluorescence of pHrodo-red zymosan measured by a microplate reader (**Figure 2E**). Next, we show that Dectin-1 engulfs intracellular zymosan particles, as verified by fluorescence profile analysis (**Figures 2F–H** and **Supplementary Figure S11)** while it was not detected at fragmented zymosan (**Figure 2G**). This implies a specific role for Dectin-1 at the early stages of zymosan recognition and uptake, rather than during intracellular processing. Collectively, our results suggest that human colonic and ileal IECs are able to phagocytose zymosan in a Dectin-1-dependent manner.

### Phagocytosis of *Candida albicans* by IECs

**Figure 3:**
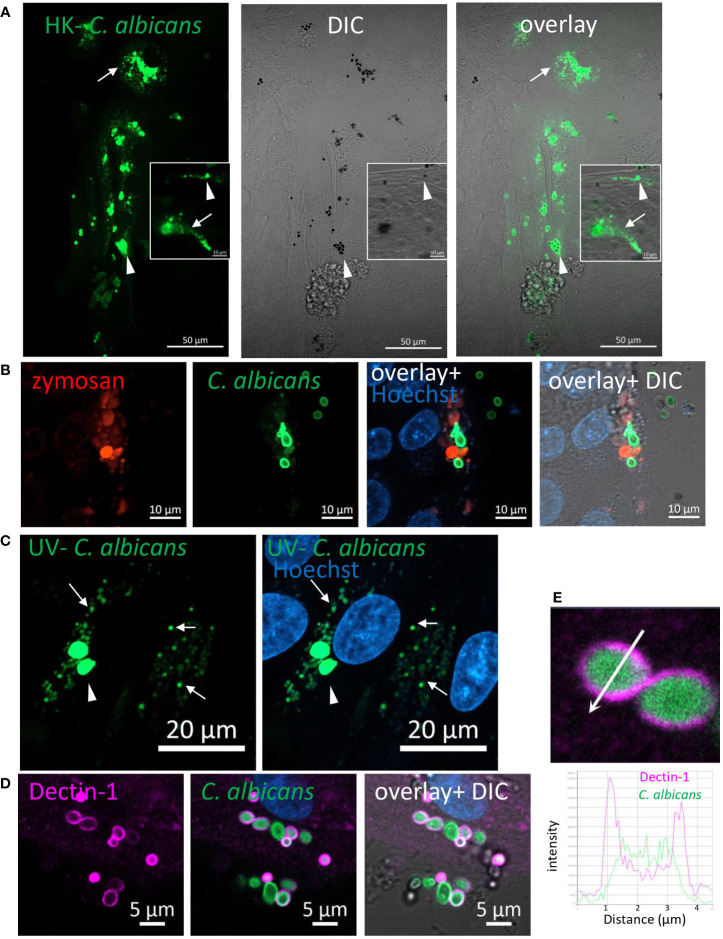
Phagocytosis of *C. albicans* by human intestinal organoids. Colonic organoids were fed overnight with Rhodamine-green-X labeled HK- *C. albicans* (**A**, green), or both pHrodo-red zymosan and HK-*C. albicans* **(B)** or UV-inactivated *C. albicans* **(C)**. Live confocal images were acquired directly or after nuclear stain with Hoechst 33342 (blue). Arrowhead - intact *C. albicans*, arrow- fragmented *C. albicans*. Original magnification x40, scale bars 50 µm **(A)**, 10 µm (**A**-inset and **B**) and 20 µm **(C)**. **(D)** Dectin-1 is recruited to phagocytosed *C. albicans*. Ileal organoids were fed with Rhodamine-green-X labeled HK- *C. albicans*. Following fixation organoids were stained with Dectin-1 polyclonal antibody. Original magnification x63, scale bar 5 µm. **(E)** Fluorescence intensity profile along the arrow of an inset from **(D)** is shown on the graph.

We next asked whether IECs phagocytose fungal particles. *C. albicans*, in its yeast-form, is a frequent commensal in the human gastrointestinal tract (25, 26). The cell-wall inner β-glucan layer is exposed in heat-killed (HK) *C. albicans*, rendering it highly accessible to Dectin-1. Yet, unlike zymosan, HK- *C. albicans* did not induce cytokine secretion by IECs, although it did elicit Syk and ERK activation (10). We therefore labeled a commercially available preparation of HK- *C. albicans* strain (ATCC 10231) with Rhodamine-green-X to assess microscopically its uptake by IECs. Colonic and ileal organoids, as well as IEC lines internalized HK- *C. albicans* particles where cellular fragmentation verified their intracellular localization and processing (**Figure 3A**; **Supplementary Figure S12A**). As observed for zymosan, here too, goblet and non-goblet cells (MUC2^+^ and MUC2^-^ respectively) phagocytosed HK- *C. albicans* (**Supplementary Figure S13**; **Figure S14**). Simultaneous exposure of organoid cultures to HK-*C. albicans* and zymosan, revealed double-labeled cells, indicating phagocytosis of both types of particles (**Figure 3B**, **Supplementary Figure S13**). Upon UV-inactivation, *C. albicans* retains its fungal cell wall intact, as in intestinal colonizing cells (27, 28). Uptake of labeled UV-inactivated *C. albicans* (of a different wild type strain-SC5314) by ileal and colonic organoids was observed, suggesting their capability to phagocytose luminal fungi (**Figure 3C**; **Supplementary Figure S12B**). Finally, Dectin-1 localization around intracellular HK-*C. albicans* (**Figure 3D**), which is supported by intensity fluorescence profile (**Figure 3E**), infers its involvement in phagocytosis. We cannot exclude the possibility that other phagocytic receptors may contribute to epithelial phagocytosis of *C. albicans*, hence, we asked whether such receptors are expressed by IECs. To this end, we tested Dectin-2/CLEC6A, which is implicated in fungal phagocytosis (29–31). Indeed, we detected Dectin-2 on the surface of human ileal and colonic IECs isolated from surgical specimens by flow cytometry (**Supplementary Figures S15A–C**), and in colonic frozen sections (**Supplementary Figure S15D**) as well. This finding further supports the idea of epithelial capability to phagocytose luminal fungi although the role of Dectin-2 in phagocytosis was not addressed here.

### LC3 is recruited to phagosomes of fungal particles

**Figure 4:**
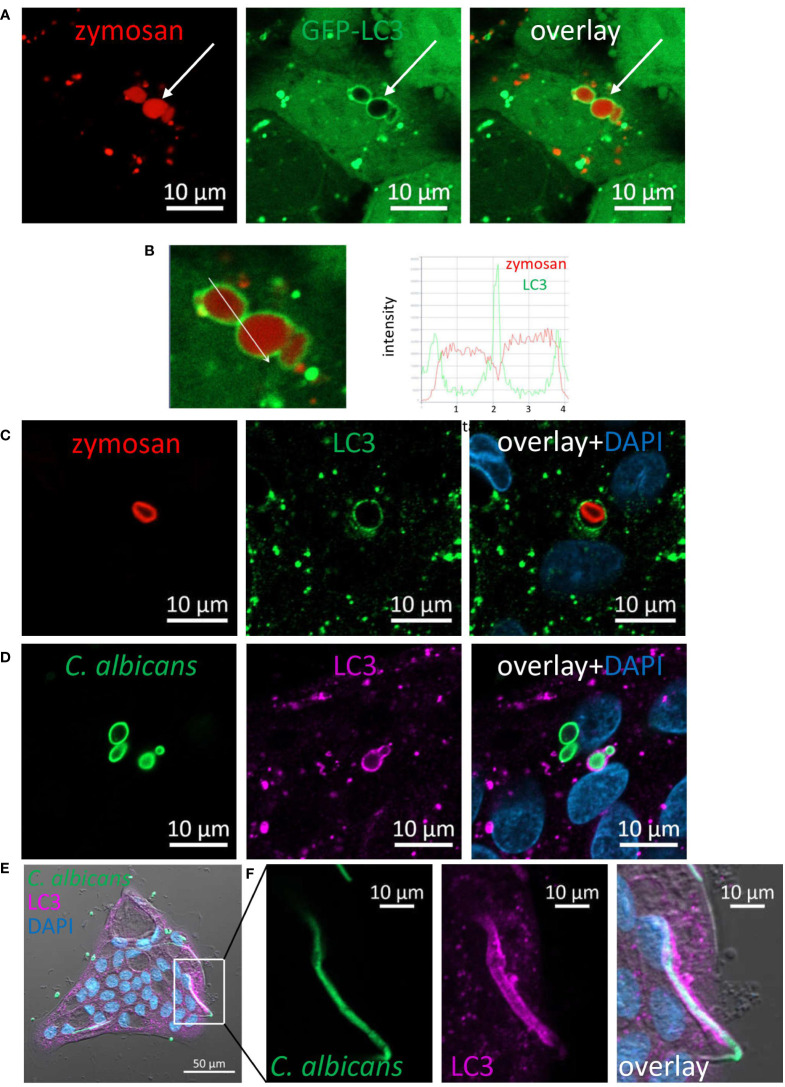
LC3 is recruited to phagosomes in IECs. **(A)** SW480 LC3-GFP cells were fed with pHrodo-red zymosan overnight. Live imaging shows LAPosomes (arrow) as LC3 (green) around intact zymosan (red) particles, as well as fragmented zymosan and autophagosomes. **(B)** Fluorescence intensity profile along the arrow of an inset from **(A)** is shown on the graph. **(C)** Colonic organoids were fed with pHrodo-red zymosan (red) overnight and stained with LC3 antibody (green) and DAPI (blue). **(D–F)** Colonic organoids were fed with Rhodamine-green-X HK-*C. albicans* (green) overnight and stained with LC3 antibody (magenta) and DAPI (blue). Shown is LAP of yeast **(D)** and hyphal form **(E, F)** of HK-*C. albicans*. F is an inset of **(E)** Original magnification ×40 **(A, B)**, ×63 **(C, D)** x20 **(E, F)** scale bar 10 µm **(A–D, F)** and 50 µm **(E)**.

LC3 associated phagocytosis (LAP) is a receptor-mediated phagocytosis that utilizes some components of the autophagy machinery to process extracellular cargo (32–34). Dectin-1 mediated LAP has been demonstrated in phagocytosis of fungi by professional phagocytes such as macrophages and dendritic cells (20, 35). To determine whether LAP of fungal particles occurs in IECs, we generated SW480 cells stably expressing GFP-tagged-LC3. These cells were incubated with pHrodo-red zymosan and using live confocal imaging pHrodo-red zymosan (**Figure 4A**) engulfed by GFP-LC3 was detected, and was further confirmed upon analysis of fluorescence intensity profile (**Figure 4B**). Similarly, using an antibody specific to LC3 LAP-engaged phagosomes (LAPosomes, (36)) of zymosan and HK-*C. albicans* in organoids (**Figures 4C, D**; **Supplementary Figure S16A**) and IEC lines were identified (**Supplementary Figure S16B**). Interestingly, the commercial preparate of HK-*C. albicans* contained a minor fraction of hyphae. We found that IECs could phagocytose and recruit LC3 to the hyphal form of *C. albicans* (**Figures 4E, F**). This surprising finding suggests that IECs can recognize and internalize a wide range of microbial forms.

### Rubicon is recruited to phagocytosed zymosan and *C. albicans*

**Figure 5:**
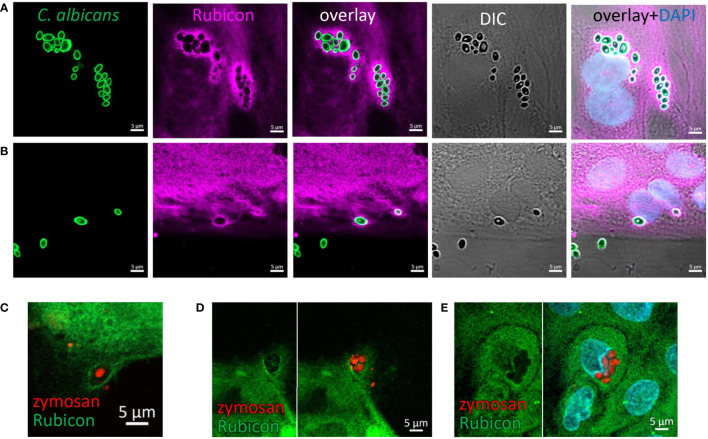
Rubicon is recruited to the phagosome. Colonic **(A)** and ileal **(B)** organoids from the same individual were fed overnight with HK-*C. albicans* (green) and stained with Rubicon antibody (magenta) and DAPI (blue). **(C–E)** Colonic **(C)** and Ileal **(D, E)** organoids were fed with pHrodo-red zymosan (red) and stained with Rubicon (green) and DAPI (cyan). Original magnification x63, scale bar 5 µm.

Rubicon is a key regulatory protein considered unique to LAP in professional phagocytes (37, 38). In order to support the notion that IECs are capable of LAP we assessed the presence of Rubicon at zymosan and *C. albicans*’ particles upon their incubation with organoids. Indeed, using a specific antibody we identified Rubicon around intracellular HK-*C. albicans* (**Figures 5A, B**) and zymosan (**Figures 5C–E**) in intestinal organoids, suggesting its recruitment and involvement in the phagocytic process, lending further support to the occurrence of LAP in IECs.

### Phagocytosis depends on NADPH-oxidase activity

**Figure 6:**
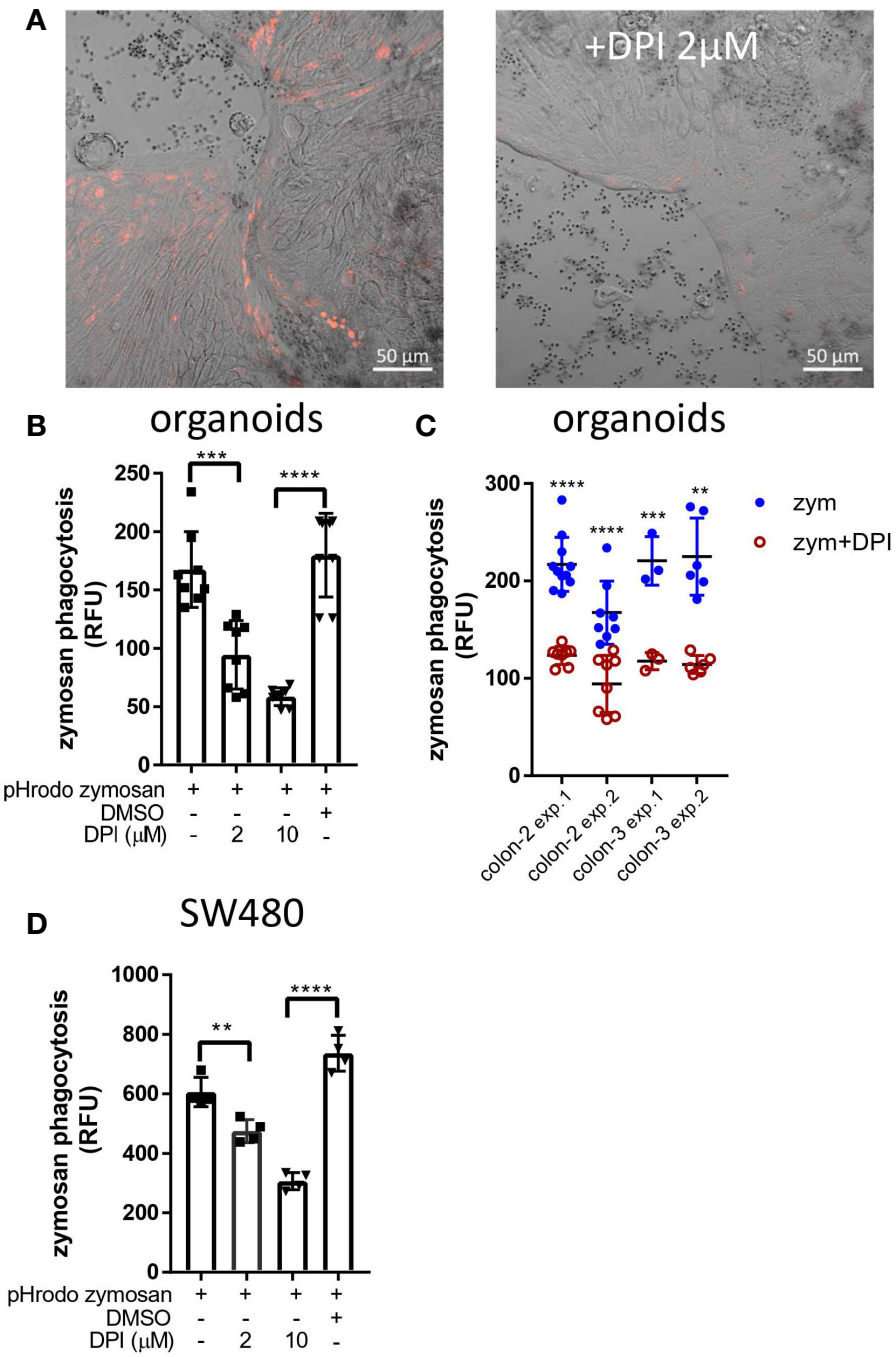
DPI inhibits zymosan phagocytosis. **(A)** Colonic organoid monolayers grown in differentiation medium for 3 days were exposed to pHrodo-red zymosan for 24 hours in the presence or absence of DPI (2 µM) that was added for 60 minutes prior to the addition of zymosan. Shown are intracellular fluorescent zymosan (red) and the organoid cells (DIC) of representative frames from 7-15 random fields imaged. The experiment was repeated 3 times using organoids from two different individuals. Original magnification x20, scale bar 50 µm. **(B)** Colonic organoids were seeded in 96 well plate, treated as in **(A)** in the presence or absence of DPI (2 or 10 µM) or in the presence of vehicle (DMSO 1:1000) in 8-replicate wells for 48 hours. Phagocytosis was assessed as the relative fluorescence by a microplate reader. **(C)** A summary of four experiments performed showing zymosan (zym) phagocytosis inhibition by 2 µM DPI in colonic organoids from two individuals (colon-2 and colon-3). **(D)** SW480 cells were seeded in 96 well plate and treated as in **(B)** in triplicates for 24 hours. Data are shown as individual measures (dots) and the mean ± SD of biological 8-replicates **(B)** and triplicates **(D)** from a representative of four or three independent experiments performed. **p<0.01, ***p<0.001, ****p<0.0001, One-way ANOVA followed by Tukey multiple comparison test **(B, D)** or individual t-test vs. no inhibitor for each of the separate experiments shown in **(C)**.

A hallmark of LAP in macrophages is the production of reactive oxygen species (ROS) by nicotinamide adenine dinucleotide phosphate (NADPH) oxidase-2 (NOX2) (33, 36). Human colonic IECs express NOX1, which is a structural homolog of NOX2 as well as additional NADPH oxidases including DUOX2 (39). To test whether NADPH-oxidases are involved in IEC-mediated phagocytosis, we treated organoid monolayers and SW480 cells with pHrodo-red zymosan after exposure to the general NADPH oxidase inhibitor diphenyleneiodonium (DPI). **Figure 6A** demonstrates that DPI (at 2 µM) drastically suppressed zymosan phagocytosis in organoids. This finding is supported by quantification of phagocytosis as reflected by total fluorescence using a microplate-reader where up to 44% and 65% inhibition by 2 µM (***p≤ 0.001) and 10 µM DPI (****p≤ 0.0001) respectively was observed (**Figure 6B**). Similar inhibition was observed in organoids from a different individual (**Figure 6C**) and in cell lines (**Figure 6D**). Our results indicate that NOX activity is necessary for phagocytosis, implying a role for ROS production and supports the occurrence of LAP in IECs.

### Phagocytosed fungi are degraded in the lysosomes

**Figure 7:**
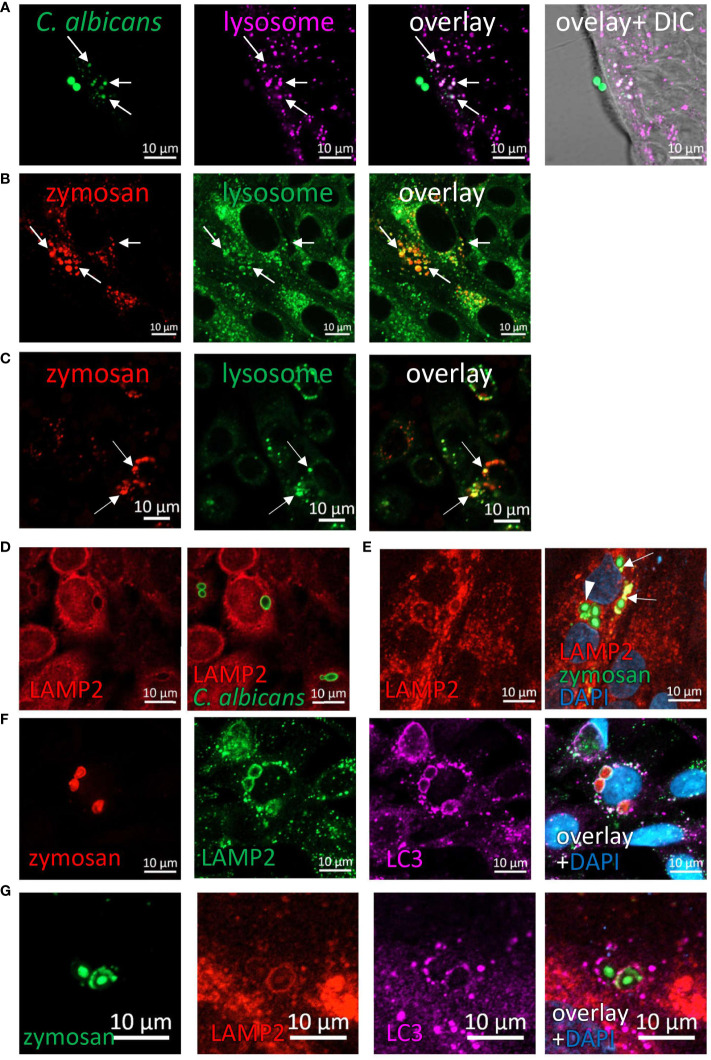
Phagocytosed particles are directed to lysosomal processing. **(A)** Colonic organoids were incubated overnight with Rhodamine-Green-X labeled HK-*C. albicans* (green) and stained with lysosomal-NIR reagent (magenta). **(B, C)** Ileal organoids **(B)** and SW480 cells **(C)** were incubated with pHrodo-red zymosan (red) and stained with lysosomal-green reagent. Arrows indicate colocalization of fragmented HK-*C. albicans* or zymosan and lysosomes. **(D)** SW480 cells were fed with HK-*C. albicans* (green) and stained with LAMP2 antibody. **(E)** Intact and fragmented zymosan particles are surrounded by LAMP2. Ileal organoids were fed with AF488-zymosan (green) overnight, and stained with LAMP2 antibody (red). Arrowhead - intact zymosan, arrow- fragmented zymosan. **(F, G)** LAPosomes merge with lysosomes. **(F)** SW480 cells were fed with pHrodo red zymosan (red) and stained with LAMP2 (green) and LC3 (magenta) antibodies and counterstained with DAPI (blue). **(G)** Ileal organoids were fed with AF488-zymosan (green) and stained with LAMP2 (red) and LC3 (magenta) antibodies and counterstained with DAPI (blue). Original magnification x63 **(A, D–G)**, x40 **(B)**, x20 **(C)**, scale bar 10 µm.

The evidence regarding intracellular fragmentation of zymosan and *C. albicans* (**Figures 1**–**3**), suggest the occurrence in of phagosome maturation *via* fusion with lysosomes and lysosomal degradation of phagosome content. To verify this, we asked whether phagocytosed zymosan and *C. albicans* colocalize with lysosomes. Indeed, intact and fragmented zymosan and *C. albicans* (both HK- and UV- inactivated) colocalized with acidic organelles as identified by lysosomal dyes in ileal and colonic organoids and in cell lines (**Figures 7A–C**; **Supplementary Figures S17A, B**). In addition, the lysosomal protein LAMP2 encircled phagocytosed particles in organoids and cell lines (**Figures 7D, E**; **Supplementary Figure S17C**). Finally, colocalization of LAMP2 with LC3 around intracellular zymosan indicates LAPosomes that fuse with lysosomes (**Figures 7F, G**).

**Figure 8:**
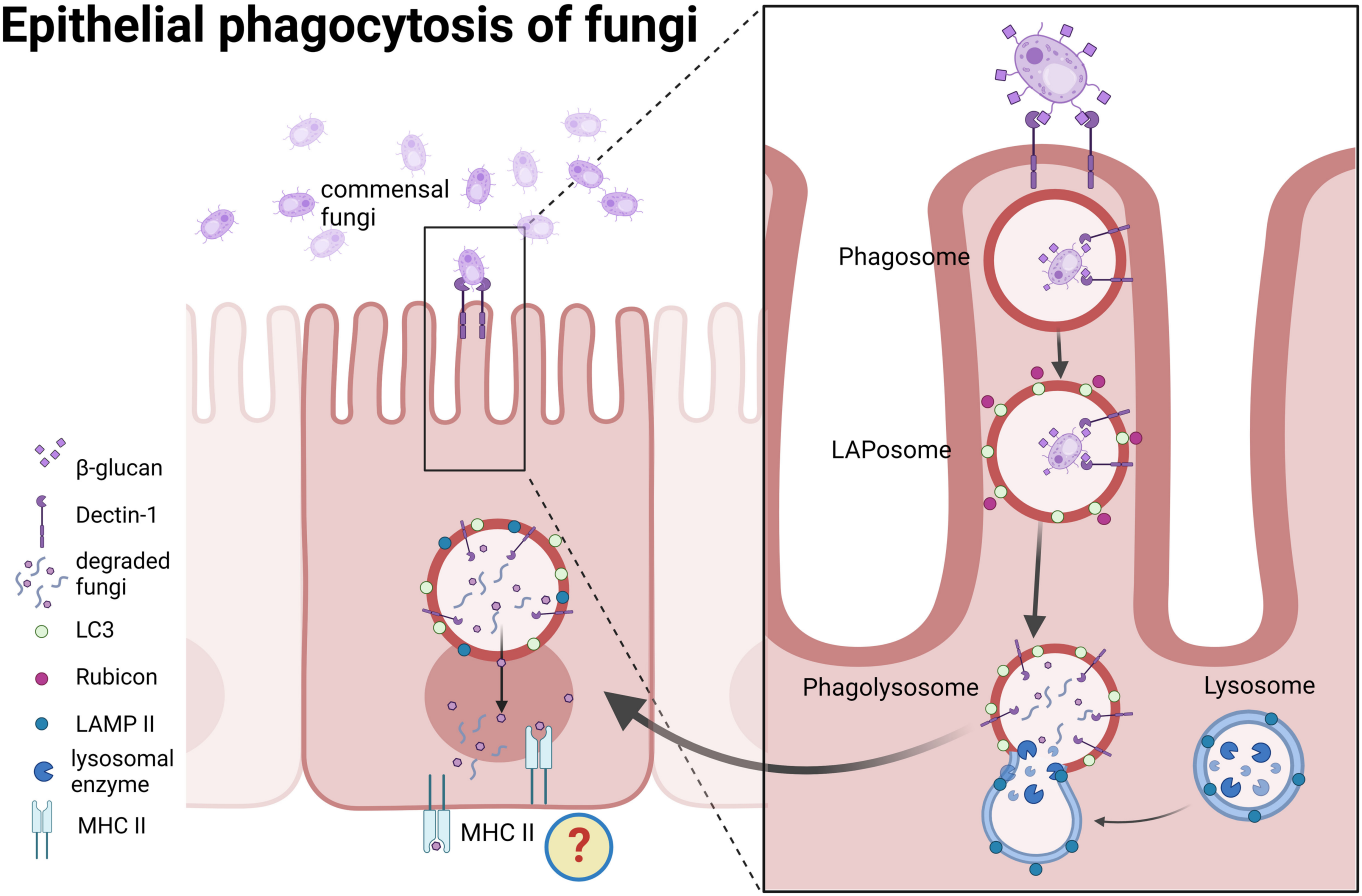
A proposed model for epithelial phagocytosis of fungi. Recognition of commensal fungi by intestinal epithelial cells leads to Dectin-1 mediated phagocytosis. Rubicon and LC3 are recruited to the phagosome and form LAPosomes. Upon fusion with lysosomes fungi are degraded. We propose that degradation products might be presented in the context of MHC class II.

Together, our findings suggest that human IECs are capable of LAP of fungal particles, and provide mechanistic evidence for stages of the process, starting from their identification by the receptor, Dectin-1, *via* recruitment of Rubicon and LC3 to final degradation in the lysosomes (**Figure 8**).

## Discussion

The intestinal epithelium acts as a physical and functional barrier as well as an active participant in mucosal immunity by orchestrating protection against pathogens and maintaining tissue homeostasis (2, 3). We and others had previously shown that IECs exert various responses upon interaction with bacterial and fungal components through pattern-recognition receptors such as Toll-like receptors (TLRs) and Dectin-1, yet they appear to tolerate various commensal microorganisms (4, 10, 40–42).

In this work we present a novel mode of IEC-microbiota interaction where IECs along the lower human gastrointestinal tract can internalize commensal fungal particles *via* LAP. Importantly, our data demonstrate that this is a host-driven process, since the phagocytosed particles are fully inactive. As non-professional phagocytes, the phagocytic capability of epithelial cells is considered limited compared to professional phagocytes (2, 43). However, their abundance on large mucosal surfaces may contribute to tissue homeostasis and the defense against pathogens (2). Indeed, previous studies in human and murine models demonstrated that epithelial cells (colonic, mammary and hair follicular) engulf apoptotic cells (44–46). Moreover, Retinal pigment epithelium used LAP to clear photoreceptor outer segments in mice (47, 48) and HeLa cells used a host derived LAP-like mechanism to target *Yersinia Pseudotuberculosis* (49).

In this work we focused on fungal recognition by IECs predominantly *via* the interaction of Dectin-1 with fungal β-glucan. However, additional phagocytic receptors such as Dectin-2 (this report) and TLRs (42) are expressed by IECs and a previous work further demonstrated TLR4 mediated bacterial phagocytosis by mouse enterocytes (50). Together these findings support the notion of epithelial capability to phagocytose luminal microorganisms.

LAP of fungi and fungal materials has been characterized in professional phagocytes (51–53) such as mouse macrophages and dendritic cells (20, 35) and in human monocytes (19). The role of Dectin-1 in this process was demonstrated by the inhibition of LAP upon Dectin-1 deficiency or following exposure to its antagonist, laminarin (19, 20). Likewise, we demonstrate in this work that Dectin-1 was involved in zymosan phagocytosis by human IECs. While Syk is activated by zymosan and fungi in IECs (4, 10, 22) it seems dispensable for phagocytosis. Examples for Dectin-1 mediated Syk-independent phagocytosis exist also in macrophages (13, 21).

Recruitment of LC3 and Rubicon, that acts as a regulatory switch to inhibit autophagy and to promote LAP (37, 38), to the internalized zymosan and *C. albicans* and the dependence of phagocytosis on ROS production are indicative for LAP. In macrophages, the phagocytic NOX2 complex plays a role in LAP, but not in canonical autophagy (33). In IECs, it might be replaced by other NADPH-oxidases, such as NOX1. While there is scarce evidence for fungal phagocytosis by epithelial cells: Dectin-1 mediated phagocytosis of spores of *Aspergillus fumigatus* in airway epithelium (54), and zymosan internalization by chicken IECs (55) we present here the first evidence for LAP in primary human epithelial cells.

Luminal sampling at steady state is important for homeostasis and building mucosal tolerance towards commensal microorganisms and is usually attributed to professional mucosal-resident phagocytes and to transcytosis (uptake and delivery without intracellular degradation) by specific epithelial M-cells (34, 56–59). The phagocytic capacity presented in this work is not likely to be assigned to M-cells for several reasons: first, it is observed in epithelial cell lines and in organoids derived from different parts of the gastrointestinal tract from ileum to sigmoid colon, while M-cells are mostly found in the small intestine and require specific differentiation conditions including TNFα and RANKL (60–62) that were not used in this study. Second, phagocytosis occurs by goblet and non-goblet cells. Finally, we find Dectin-1 mediated LAP, and epithelial processing of the internalized particle, as opposed to the transcytosis of intact particles by M-cells.

The role of commensal fungi in shaping mucosal tolerance and host systemic immune response has been recently established in a series of reports (26, 63–65) underlying their interaction with mucosal immune cells e.g., lymphocytes *via* mononuclear phagocytes. Here we propose epithelial phagocytosis as another pathway for intestinal mucosal sensing of fungi. While the physiological outcome of epithelial phagocytosis of fungi is still uncovered, it is plausible to assume that it has an impact on the mucosal milieu. Indeed, an example where IECs acquire antigens from commensal bacteria (segmented filamentous bacteria, SFB), for generation of TH17 cell responses in mice was recently provided (41). IECs endocytosed vesicles containing SFB cell wall–associated proteins, that acted as immunomodulators on T cells. Still, the mechanism by which IECs induced Th17 differentiation remains unclear. Interestingly, mucosa-associated fungi elicit a protective effect on IECs barrier function and protected mice against intestinal injury and bacterial infection *via* Th17 cells (65). It is not known whether IECs play an active role in the observed increased frequency of Th17 cells.

In professional phagocytes, LAP of fungi enhances pathogen killing (35) (19), cytokine secretion (30)and promotes MHC class II recruitment to the LAPosome for sustained antigen presentation (20).

IECs express MHC class II and co-stimulatory molecules and hence were suggested to act as antigen presenting cells (APCs) (66–69). Indeed, there is evidence that MHC class II-expressing IECs functioned as APCs to prime donor CD4+ T cells *ex-vivo* and *in vivo* where microbiota influences MHC class II expression on IECs in the ileum. Still, the presented peptides were mostly thought as endogenous peptides (70). Whether IECs can present exogenous peptide is not clear, and an experimental model was presented to study the interactions between IEC MHC-II and the surrounding immune and microbial milieu (71). We propose that degradation products of phagocytosed fungi might be presented in context with MHC class II by IECs, similarly to their presentation upon phagocytosis by professional antigen presenting cells (**Figure 8**). In this case, IECs may contribute to humoral responses. It was recently found that commensal fungi induce, *via* intestinal mononuclear phagocytes, the production of secretory IgA (sIgA) in the murine gut and systemic serum IgG (63, 64). A decrease in antifungal sIgA was observed in patients with Crohn’s disease (64), underlying a feature of loss of tolerance towards commensal microorganisms, which is typical of inflammatory bowel diseases (IBD) (72). Accordingly, possible contribution of interrupted epithelial LAP to disrupted mucosal homeostasis and loss of tolerance may be assumed. In our hands, IBD derived organoids could phagocytose fungal particles *via* LAP in a similar manner to the healthy ones. Yet this is a small cohort, and an interrupted LAP is expected to be detected in organoids if it is carried genetically or under inflammatory conditions. An attractive approach will be to assess whether genes associated with IBD that may function in LAP indeed affect epithelial LAP. An immediate candidate from our work is NOX1, which has recently been shown to prevent inflammation, and its mutations were linked to ulcerative colitis (39, 62). Another interesting candidate is the ATG16L1 T300A polymorphism. Individuals carrying this polymorphism exhibit defects in T-regulatory responses to outer-membrane-vesicles (OMVs) of the commensal *Bacteroides fragilis*, and sensing those OMVs, in mouse dendritic cells, occurs *via* LAP and involves Rubicon and ATG16L1 (58).

Our findings may have mechanistic and translational implications, as well as paving the way for detailed characterization of the processes underlying epithelial phagocytosis of microorganisms. Specifically, facilitating further identification of host and microbial factors, such as cell wall composition of pathogens compared to commensals, that control epithelial phagocytosis. Our experimental setting may also be useful to study the outcome of IECs’ phagocytosis with respect to the impact on neighboring cells (e.g., T-cells). Using patient derived organoids (73), our findings may allow the assessment of epithelial phagocytic capabilities under defined genetic (e.g. ATG16L1 variants) and experimental inflammatory conditions and evaluate their contribution to homeostasis or if perturbed, to the pathogenesis of IBD.

## Methods

### Cell lines

Human colon epithelial cell lines SW480, HCT116 and Caco-2 were purchased from ATCC (Manassas, VA). SW480 and HCT116 cells were grown in RPMI medium (01-100-1A, Biological Industries) supplemented with 10% fetal bovine serum (04-007-1A, Biological Industries), and Caco-2 cells were grown in EMEM (01-040-1A, Biological Industries), supplemented with 20% fetal bovine serum. All growth media contained 100 units/mL penicillin G, and 100 µg/mL streptomycin (03-031-1B, Biological Industries). All cells were maintained in a humidified incubator at 37°C with 5% CO_2_.

To generate cells stably expressing GFP-LC3, SW480 cells were transfected with pEGFP-LC3 using the Lipofectamine 2000 reagent (Invitrogen) according to the manufacturer’s instructions. Stable clones expressing GFP-LC3 were selected and cultured in the presence of 1000 μg/ml geneticin (G-418, 345810, Calbiochem). pEGFP-LC3 (human) was a kind gift from Toren Finkel (74) (Addgene plasmid # 24920).

### Human samples and ethics statement

The Institutional Ethical Committee of the Rabin Medical Center approved the study (approval number 0763-16-RMC and 0298-17) and a written informed consent of all participating subjects was obtained. The identity of all participating subjects remained anonymous. Tissue samples were taken from surgical specimens of patients undergoing bowel resection for colonic tumors (normal ileal or colonic samples were taken from a distance of at least 10 cm from the tumor) or patients with Crohn’s disease or ulcerative colitis undergoing bowel resection. Specimens were kept overnight at 4°C in RPMI containing 100 units/mL penicillin G (03-031-1B, Biological industries), and 100 µg/mL streptomycin and 2.5 µg/mL amphotericin B (Fungizone, 03-028-1B, Biological industries) supplemented with 10% fetal bovine serum.

### Human intestinal organoids

Human ileal and colonic crypts were isolated and organoids were cultured as previously described (75). In brief, tissue fragments were washed twice with crypt isolation medium: 0.5 mM DL-Dithiothreitol (DTT), 5.6 mM Na_2_HPO_4_, 8 mM KH_2_PO_4_, 96.2 mM NaCl, 1.6 mM KCl, 43.4 mM sucrose, 54.9 mM D-sorbitol. Tissue was incubated in crypt isolation medium supplemented with 2 mM EDTA, for 30 minutes at 4°C followed by vigorously shaking till crypt were released from the mesenchyme. Crypt pellet was washed with FBS and resuspended in ice-cold Matrigel (FAL356231, Corning) and seeded as 15 µl domes on pre-warmed 12-wells tissue culture plates. Plates were incubated upside down for 20 min in a 37°C 5% CO2 incubator until the Matrigel solidifies. Organoid expansion media [based on (76, 77)] consisted of advanced DMEM F12 (12634010, Gibco) (26% of total volume), 100/100 U/ml Penicillin/streptomycin, 10 mM HEPES (10 mM, 03-025-1B, Biological Industries), 1× GlutaMAX (35050-038, Gibco), and the following growth factors: 1× B27(12587001, Gibco), 1 mM N-Acetylcysteine (A9165, Sigma-Aldrich), 100 ng/ml Noggin (120-10C, Peprotech), 50 ng/ml human EGF (AF-100-15, Peprotech), 10 mM Nicotinamide (N0636, Sigma-Aldrich), 10 μM SB202190 (1264, Tocris), 500 nM A83-01 (2939, Tocris), 10 nM Prostaglandine E2 (2296, Tocris), 26 μg/ml Primocin (ant-pm *In vivo*Gen), and conditioned medium from the L cell line secreting Wnt3A (50% of total volume) and 293T cells secreting R-spondin 1 (20%). 10 μM Y-27632 (Rock inhibitor, Y0503, Sigma-Aldrich) was added to expansion medium for the first 2 days. Medium was changed every other day.

2D organoid monolayer culture protocol was based on the supplementary protocol of Intesticult™ medium (WWW.STEMCELL.COM) culture: µ-Slide 8-well glass bottom chambers (Ibidi, Martinsried, Germany) were coated with 1:50 Matrigel in PBS for one hour at a 37°C 5% CO2 incubator. 3D organoids were resuspended with Recombinant Trypsin EDTA Solution (03-079-1A, Biological Industries) and mechanically disrupted into a single cell suspension. Cells were resuspended in expansion medium containing 10 μM Y-27632 (Rock inhibitor) and seeded on coated slides (approximately 2 domes per 8-well chamber). Expansion medium was changed every other day until 2D organoids reached 50-70% confluency. Prior to phagocytosis experiments, 2D organoids were grown for additional 2-3 days in a generic differentiation medium based on (77). Briefly, advanced DMEM F12 was supplemented with 100/100 U/ml Penicillin/streptomycin, 10 mM HEPES, 1× GlutaMAX, 1× B27, 1 mM N-Acetylcysteine, 500 ng/ml human R-spondin 1 (120-38, Peprotech), 100 ng/ml Noggin, 50 ng/ml human EGF, and 100 μg/ml Primocin.

### *Candida albicans* growth conditions and labeling

*C. albicans* wild type strain SC5314 was kindly provided by Judith Berman (Tel Aviv University). Cells were grown overnight in YPAD medium at 30°C. UV inactivated *C. albicans* cells were prepared as previously described (78). Briefly, cells were exposed in a thin liquid suspension to 4 doses of UV radiation (100 mJ/cm^2^) in a UV cross linker (CL-1000 UVP, Upland, CA). Cells were counted and resuspended in PBS. Killing was verified by seeding onto YPAD-agar plates. Heat-killed *C. albicans* wild type strain ATCC 10231 (tlrl-hkca) was purchased from *In vivo*Gen (San Diego, CA) and resuspended in endotoxin-free water.

Heat-killed or UV-inactivated particles of *C. albicans* were labeled with Rhodamine Green-X (R-6113, Life Technologies, Invitrogen), as previously described (79). After labeling cells were washed, resuspended in PBS, counted and aliquoted.

### Confocal phagocytosis assay

IEC lines were seeded on μ 8-well glass-bottomed slides (Ibidi, Martinsried) at a density of 3-4x10^4^ cells/well. Two days later, medium was replaced and cells were stimulated overnight with pHrodo-red zymosan (100 µg/ml, P35364 Invitrogen) or AF488-zymosan (100 µg/ml) Z23373 Invitrogen) or Rhodmine-green-x labeled *C. albicans* (approximately at 0.3-1×10^6^/well). We counted about 10^5^ cells/well, hence, MOI= 3-10/cell). The next day cells were either directly imaged with confocal microscope (LSM 800, Zeiss, and Zen3.2 software) or stained with Hoechst 33342 (14533, Sigma) to visualize nuclei or with lysosomal staining reagents (Green - Cytopainter ab176826 or NIR - Cytopainter ab176824, Abcam). For image analysis and fluorescence intensity profile we used Zen 3.2 software. Where indicated, the inhibitors Cytochalasin-D (10 µM, MBS250255, Calbiochem) or laminarin (1 mg/ml, L9634, Sigma) or diphenyleneiodonium (DPI) (2 µM, D2926, Sigma) were added 1 hour prior to pHrodo-red zymosan addition.

2D organoid monolayers were seeded as described above, and grown in differentiation medium for at least 2 days prior to their exposure to labeled zymosan or *C. albicans.* Then, organoids were treated and assessed as the cell lines. In some cases, cells or organoids were fixed with 4% paraformaldehyde or 100% methanol for further immuno-staining. All images within each experiment were acquired under the same conditions.

### Image quantification of phagocytosis

We quantified phagocytosis either by confocal image analysis using ImagJ or by multi-well-plate fluorescence quantification using a microplate reader. Image-analysis quantification was applied on confocal images of cell lines, where random fields acquired were representative of the uniformly distributed phagocytic cells. For each experimental condition (phagocytosis in the presence or absence of inhibitors) the number of nuclei within at least four confocal random fields (acquired at x20 magnification) was determined with ImageJ. Then the number of pHrodo-red zymosan positive cells within each field was manually counted. The percentage of positive cells was calculated. For each condition, at least 2000 cells were analyzed.

### Microplate reader quantification of phagocytosis in cell lines and organoids

We assessed the level of phagocytosis by quantification of the total fluorescence within replicate wells as an additional assay for cell lines, and as the main assay quantify phagocytosis in organoid monolayers where it was highly important to quantify the whole well since phagocytosis is not uniformly distributed. SW480 and HCT116 cells were seeded in 96-well flat-bottomed plastic plates at a density of 4x10^4^ cells/well for 24 hours. The next day the medium was replaced with 100 µl/well of fresh medium or medium that contains inhibitors (Cytochalasin-D 10 µM, Laminarin 1 mg/ml, Syk inhibitor 574711 [3-(1-methyl-1H-indol-3-yl-methylene)-2-oxo-2,3-dihydro-1H-indole-5-sulfonamide], (Calbiochem, Merck-Millipore) 1 or 5 µM, DPI (2 or 10 µM) or DMSO as vehicle (1:1000) where applicable and pHrodo-red zymosan at 100 µg/ml was added in triplicate wells for 24 hours. Wells were washed gently 3 times with white-RPMI and relative fluorescence was measured with Synergy H1 microplate reader (Biotek). For phagocytosis quantification in organoids, 96-well plastic plates were coated with Matrigel as described for 8-well chambers. Cells from 3D human colonic organoids were resuspended and seeded in a ratio of one-two domes to 60 wells or 10,000 cells/well. Organoid monolayers were grown in expansion medium for at least 3 days, reaching at least 50% confluence, and then the medium was replaced with differentiation medium for 2-3 days (where wells were 80-90% confluent). Before stimulation, medium was replaced with fresh differentiation medium in the presence or absence of laminarin (1 mg/ml) or DPI (2 or 10 µM), and pHrodo-red zymosan at 100 µg/ml was added in triplicates to 8-replicate wells as indicated for 48 hours to allow accumulation of phagocytic cells thus obtaining enhanced phagocytosis signal. Medium was removed and organoids were washed gently 3 times with white-RPMI before relative fluorescence was assessed in a microplate reader.

### Cytokine secretion (ELISA)

SW480 cells were seeded in 96-well flat-bottomed plastic plates at 4x10^4^ cells/well for 24 hours. The next day the medium was replaced with fresh medium or medium that contains Syk inhibitor (1 or 5 µM/ml) (100 µl/well) and one hour later, zymosan (100 µg/ml, tlrl-zyn, *In vivo*Gen, not labeled) was added in triplicate wells for 24 hours. Supernatants were assessed for IL-8 secretion using ELISA (DY208 R&D systems) according to the manufacturer’s instructions. In parallel, in the same 96-well plate, triplicate wells were exposed to pHrodo-red zymosan in the presence and absence of the same Syk inhibitor, for phagocytosis microplate reader quantification assay.

### Cell staining by immunofluorescence

Cells or organoid monolayers seeded in 8-well chambers and treated as indicated in the figure legends, were fixed with 4% paraformaldehyde for 30 minutes at room temperature or with ice-cold methanol for 15 minutes at -20°C according to the requirement of the primary antibodies, washed with PBS, and blocked-permeabilized with 5% donkey serum containing 0.3% triton for 1 hour at room temperature. Cells or organoid monolayers were incubated with the indicated primary antibodies overnight at 4°C, followed by staining with the corresponding fluorescently labeled secondary antibodies for 1 hour at room temperature and counterstaining with DAPI, which was included in the non-hardening mounting medium (GBI Labs, E-19-18, Mukilteo, WA). Samples were visualized by inverted confocal microscope (LSM 800, Zeiss). All images within each experiment were acquired under the same conditions. Details of primary and secondary antibodies appear **Table 1**.

**Table 1:** Antibodies used for immuno-fluorescence.

Antibody	host	Fixation	Dilution	Cat. #	Company
Dectin-1	Rabbit polyclonal	Methanol	1:200	NBP1–25514	ovus Biologicals
Dectin-1 (GE2)	Mouse monoclonal	PFA	1:50	ab82888	Abcam
LC3A/B (D3U4C)	Rabbit monoclonal	Methanol	1:100	12741	Cell Signaling
Rubicon	Mouse monoclonal	PFA	1:100	ab156052	Abcam
LAMP2 (H4B4)	Mouse monoclonal	Methanol	1:200	sc-18822	Santa cruz
Ki67	Rabbit monoclonal	PFA	1:400	RBK027	Zytomed
Muc2 (996/1)	Mouse monoclonal	PFA	1:200	ab11197	Abcam
Lysozyme	Rabbit polyclonal	PFA	1:400	A 0099	Dako
Dectin-2	Mouse monoclonal	PFA	1:100	ab107572	Abcam
ZO-1/TJP1	Mouse monoclonal	PFA	1:100	33-9100	Thermo Scientific
EpCAM (VU-1D9)	Mouse monoclonal	PFA or Methanol	1:400	187372	Abcam
EpCAM	Rabbit polyclonal	PFA or Methanol	1:400	ab71916	Abcam
anti mouse AF488	Donkey		1:1000	ab150109	Abcam
anti mouse AF647	Donkey		1:1000	ab216773	Abcam
anti rabbit AF488	Donkey		1:1000	ab150065	Abcam
anti rabbit AF647	Donkey		1:1000	ab150067	Abcam

### Statistical analysis

Cell line results are reported as the measured data point and the mean of triplicates ± SD of a representative of at least 3 experiments performed. Organoid quantitative data are represented as the summary of the experiments performed in separate organoids. Significance was determined using unpaired two-tailed student’s *t*-test or one-step ANOVA as indicated (GraphPad Prism 7.03 software, San Diego, CA). Differences were noted as significant by the following conventions: *p<0.05; **p<0.01; ***p<0.001 ****p<0.0001, as specifically indicated in the legend of each figure.

## Data availability statement

The raw data supporting the conclusions of this article will be made available by the authors, without undue reservation.

## Ethics statement

The studies involving human participants were reviewed and approved by The Institutional Ethical Committee of the Rabin Medical Center (approval number 0763-16-RMC and 0298-17). The patients/participants provided their written informed consent to participate in this study.

## Author contributions

SC-K and ID contributed to the conception and design of the study, and wrote the manuscript. SC-K performed the experiments and prepared the figures. SC-K, LB and KK performed the statistical analysis. ESB and KMR generated the organoids, ESB, KMR, DK, HA-T, SS, KK and LB assisted in all experiments, data acquisition, data interpretation and critical advice throughout the project. ES, IW, NW, MW-B and AL-B assisted in collecting human samples. ID obtained funding. All authors contributed to the article and approved the submitted version.

## References

[1] Allaire JM Crowley SM Law HT Chang S-Y Ko H-J Vallance BA. The intestinal epithelium: Central coordinator of mucosal immunity. Trends Immunol (2018) 39:677–96. doi: 10.1016/j.it.2018.04.002 29716793

[2] Gunther J Seyfert HM. The first line of defence: insights into mechanisms and relevance of phagocytosis in epithelial cells. Semin Immunopathol (2018) 40:555–65. doi: 10.1007/s00281-018-0701-1 PMC6223882 30182191

[3] Soderholm AT Pedicord VA. Intestinal epithelial cells: at the interface of the microbiota and mucosal immunity. Immunology (2019) 158:267–80. doi: 10.1111/imm.13117 PMC6856932 31509239

[4] Cohen-Kedar S Baram L Elad H Brazowski E Guzner-Gur H Dotan I. Human intestinal epithelial cells respond to beta-glucans *via* dectin-1 and syk. Eur J Immunol (2014) 44:3729–40. doi: 10.1002/eji.201444876 25251945

[5] Del Fresno C Iborra S Saz-Leal P Martinez-Lopez M Sancho D. Flexible signaling of myeloid c-type lectin receptors in immunity and inflammation. Front Immunol (2018) 9:804. doi: 10.3389/fimmu.2018.00804 29755458 PMC5932189

[6] Salazar F Brown GD. Antifungal innate immunity: A perspective from the last 10 years. J Innate Immun (2018) 10:373–97. doi: 10.1159/000488539 PMC6784043 29768268

[7] Höft MA Hoving JC Brown GD. Signaling c-type lectin receptors in antifungal immunity. Curr topics Microbiol Immunol (2020) 429:63–101. doi: 10.1007/82_2020_224 32936383

[8] Nikolakopoulou C Willment JA Brown GD. C-type lectin receptors in antifungal immunity. Adv Exp Med Biol (2020) 1204:1–30. doi: 10.1007/978-981-15-1580-4_1 32152941

[9] Mata-Martínez P Bergón-Gutiérrez M del Fresno C. Dectin-1 signaling update: New perspectives for trained immunity. Front Immunol (2022) 13. doi: 10.3389/fimmu.2022.812148 PMC8882614 35237264

[10] Cohen-Kedar S Keizer D Schwartz S Rabinowitz KM Kaboub K Shaham Barda E. Commensal fungi and their cell-wall beta-glucans direct differential responses in human intestinal epithelial cells. Eur J Immunol (2021) 51:864–78. doi: 10.1002/eji.202048852 33616974

[11] Brown GD Gordon S. Immune recognition. a new receptor for beta-glucans. Nature (2001) 413:36–7. doi: 10.1038/35092620 11544516

[12] Taylor PR Tsoni SV Willment JA Dennehy KM Rosas M Findon H. Dectin-1 is required for beta-glucan recognition and control of fungal infection. Nat Immunol (2007) 8:31–8. doi: 10.1038/ni1408 PMC1888731 17159984

[13] Herre J Marshall AS Caron E Edwards AD Williams DL Schweighoffer E. Dectin-1 uses novel mechanisms for yeast phagocytosis in macrophages. Blood (2004) 104:4038–45. doi: 10.1182/blood-2004-03-1140 15304394

[14] Gantner BN Simmons RM Underhill DM. Dectin-1 mediates macrophage recognition of candida albicans yeast but not filaments. EMBO J (2005) 24:1277–86. doi: 10.1038/sj.emboj.7600594 PMC556398 15729357

[15] Lindner B Burkard T Schuler M. Phagocytosis assays with different pH-sensitive fluorescent particles and various readouts. Biotechniques (2020) 68:245–50. doi: 10.2144/btn-2020-0003 32079414

[16] Luther K Torosantucci A Brakhage AA Heesemann J Ebel F. Phagocytosis of aspergillus fumigatus conidia by murine macrophages involves recognition by the dectin-1 beta-glucan receptor and toll-like receptor 2. Cell Microbiol (2007) 9:368–81. doi: 10.1111/j.1462-5822.2006.00796.x 16953804

[17] Fuentes AL Millis L Sigola LB. Laminarin, a soluble beta-glucan, inhibits macrophage phagocytosis of zymosan but has no effect on lipopolysaccharide mediated augmentation of phagocytosis. Int Immunopharmacol (2011) 11:1939–45. doi: 10.1016/j.intimp.2011.08.005 21856445

[18] Maneu V Yáñez A Murciano C Molina A Gil ML Gozalbo D. Dectin-1 mediates *in vitro* phagocytosis of candida albicans yeast cells by retinal microglia. FEMS Immunol Med Microbiol (2011) 63:148–50. doi: 10.1111/j.1574-695X.2011.00829.x 21668824

[19] Kyrmizi I Gresnigt MS Akoumianaki T Samonis G Sidiropoulos P Boumpas D. Corticosteroids block autophagy protein recruitment in aspergillus fumigatus phagosomes *via* targeting dectin-1/Syk kinase signaling. J Immunol (2013) 191:1287–99. doi: 10.4049/jimmunol.1300132 PMC3883106 23817424

[20] Ma J Becker C Lowell CA Underhill DM. Dectin-1-triggered recruitment of light chain 3 protein to phagosomes facilitates major histocompatibility complex class II presentation of fungal-derived antigens. J Biol Chem (2012) 287:34149–56. doi: 10.1074/jbc.M112.382812 PMC3464523 22902620

[21] Underhill DM Rossnagle E Lowell CA Simmons RM. Dectin-1 activates syk tyrosine kinase in a dynamic subset of macrophages for reactive oxygen production. Blood (2005) 106:2543–50. doi: 10.1182/blood-2005-03-1239 PMC1895265 15956283

[22] Wang L Aschenbrenner D Zeng Z Cao X Mayr D Mehta M. Gain-of-function variants in SYK cause immune dysregulation and systemic inflammation in humans and mice. . Nat Genet (2021) 53:500–10. doi: 10.1038/s41588-021-00803-4 PMC8245161 33782605

[23] Dutta D Heo I Clevers H. Disease modeling in stem cell-derived 3D organoid systems. Trends Mol Med (2017) 23:393–410. doi: 10.1016/j.molmed.2017.02.007 28341301

[24] Thorne CA Chen IW Sanman LE Cobb MH Wu LF Altschuler SJ. Enteroid monolayers reveal an autonomous WNT and BMP circuit controlling intestinal epithelial growth and organization. Dev Cell (2018) 44:624–633.e624. doi: 10.1016/j.devcel.2018.01.024 29503158 PMC5849535

[25] Sokol H Leducq V Aschard H Pham HP Jegou S Landman C. Fungal microbiota dysbiosis in IBD. Gut (2017) 66:1039–48. doi: 10.1136/gutjnl-2015-310746 PMC5532459 26843508

[26] Li XV Leonardi I Putzel GG Semon A Fiers WD Kusakabe T. Immune regulation by fungal strain diversity in inflammatory bowel disease. Nature (2022) 603:672–8. doi: 10.1038/s41586-022-04502-w PMC9166917 35296857

[27] Galan-Diez M Arana DM Serrano-Gomez D Kremer L Casasnovas JM Ortega M. Candida albicans beta-glucan exposure is controlled by the fungal CEK1-mediated mitogen-activated protein kinase pathway that modulates immune responses triggered through dectin-1. Infect Immun (2010) 78:1426–36. doi: 10.1128/IAI.00989-09 PMC2849429 20100861

[28] Esteban A Popp MW Vyas VK Strijbis K Ploegh HL Fink GR. Fungal recognition is mediated by the association of dectin-1 and galectin-3 in macrophages. Proc Natl Acad Sci USA (2011) 108:14270–5. doi: 10.1073/pnas.111141510 PMC3161568 21825168

[29] Ifrim DC Quintin J Courjol F Verschueren I van Krieken JH Koentgen F. The role of dectin-2 for host defense against disseminated candidiasis. J Interferon Cytokine Res (2016) 36:267–76. doi: 10.1089/jir.2015.0040 PMC4827303 27046240

[30] Lamprinaki D Beasy G Zhekova A Wittmann A James S Dicks J. LC3-associated phagocytosis is required for dendritic cell inflammatory cytokine response to gut commensal yeast saccharomyces cerevisiae. Front Immunol (2017) 8. doi: 10.3389/fimmu.2017.01397 PMC5661120 29118762

[31] Kitai Y Sato K Tanno D Yuan X Umeki A Kasamatsu J. Role of dectin-2 in the phagocytosis of cryptococcus neoformans by dendritic cells. Infection Immun (2021) 89:e00330–00321. doi: 10.1128/IAI.00330-21 PMC8445189 34251289

[32] Sanjuan MA Dillon CP Tait SW Moshiach S Dorsey F Connell S. Toll-like receptor signalling in macrophages links the autophagy pathway to phagocytosis. Nature (2007) 450:1253–7. doi: 10.1038/nature06421 18097414

[33] Heckmann BL Green DR. LC3-associated phagocytosis at a glance. J Cell Sci (2019) 132(5):jcs222984. doi: 10.1242/jcs.222984 30787029 PMC6432721

[34] Grijmans BJM van der Kooij SB Varela M Meijer AH. LAPped in proof: LC3-associated phagocytosis and the arms race against bacterial pathogens. Front Cell Infect Microbiol (2022) 11. doi: 10.3389/fcimb.2021.809121 PMC8762105 35047422

[35] Tam JM Mansour MK Khan NS Seward M Puranam S Tanne A. Dectin-1-dependent LC3 recruitment to phagosomes enhances fungicidal activity in macrophages. J Infect Dis (2014) 210:1844–54. doi: 10.1093/infdis/jiu290 PMC4271056 24842831

[36] Martinez J Malireddi RK Lu Q Cunha LD Pelletier S Gingras S. Molecular characterization of LC3-associated phagocytosis reveals distinct roles for Rubicon, NOX2 and autophagy proteins. Nat Cell Biol (2015) 17:893–906. doi: 10.1038/ncb3192 26098576 PMC4612372

[37] Boyle KB Randow F. Rubicon Swaps autophagy for LAP. Nat Cell Biol (2015) 17:843–5. doi: 10.1038/ncb3197 26123110

[38] Wong SW Sil P Martinez J. Rubicon: LC3-associated phagocytosis and beyond. FEBS J (2018) 285:1379–88. doi: 10.1111/febs.14354 PMC6779045 29215797

[39] Schwerd T Bryant RV Pandey S Capitani M Meran L Cazier JB. NOX1 loss-of-function genetic variants in patients with inflammatory bowel disease. Mucosal Immunol (2018) 11:562–74. doi: 10.1038/mi.2017.74 PMC5924597 29091079

[40] Richardson JP Ho J Naglik JR. Candida–epithelial interactions. J Fungi (2018) 4:22. doi: 10.3390/jof4010022 PMC5872325 29419738

[41] Ladinsky MS Araujo LP Zhang X Veltri J Galan-Diez M Soualhi S. Endocytosis of commensal antigens by intestinal epithelial cells regulates mucosal T cell homeostasis. Science (2019) 363(6431):eaat4042. doi: 10.1126/science.aat4042 30846568 PMC6708280

[42] Burgueño JF Abreu MT. Epithelial toll-like receptors and their role in gut homeostasis and disease. Nat Rev Gastroenterol Hepatol (2020) 17:263–78. doi: 10.1038/s41575-019-0261-4 32103203

[43] Freeman SA Grinstein S. Phagocytosis: How macrophages tune their non-professional counterparts. Curr Biol (2016) 26:R1279–82. doi: 10.1016/j.cub.2016.10.059 27997839

[44] Mesa KR Rompolas P Zito G Myung P Sun TY Brown S. Niche-induced cell death and epithelial phagocytosis regulate hair follicle stem cell pool. Nature (2015) 522:94–7. doi: 10.1038/nature14306 PMC4457634 25849774

[45] Fornetti J Flanders KC Henson PM Tan AC Borges VF Schedin P. Mammary epithelial cell phagocytosis downstream of TGF-beta3 is characterized by adherens junction reorganization. Cell Death Differ (2016) 23:185–96. doi: 10.1038/cdd.2015.82 PMC4716300 26113040

[46] Lee CS Penberthy KK Wheeler KM Juncadella IJ Vandenabeele P Lysiak JJ. Boosting apoptotic cell clearance by colonic epithelial cells attenuates inflammation *In vivo* . Immunity (2016) 44:807–20. doi: 10.1016/j.immuni.2016.02.005 PMC4838559 27037190

[47] Kim JY Zhao H Martinez J Doggett TA Kolesnikov AV Tang PH. Noncanonical autophagy promotes the visual cycle. Cell (2013) 154:365–76. doi: 10.1016/j.cell.2013.06.012 PMC3744125 23870125

[48] Muniz-Feliciano L Doggett TA Zhou Z Ferguson TA. RUBCN/rubicon and EGFR regulate lysosomal degradative processes in the retinal pigment epithelium (RPE) of the eye. Autophagy (2017) 13:2072–85. doi: 10.1080/15548627.2017.1380124 PMC5788552 28933590

[49] Ligeon LA Moreau K Barois N Bongiovanni A Lacorre DA Werkmeister E. Role of VAMP3 and VAMP7 in the commitment of yersinia pseudotuberculosis to LC3-associated pathways involving single- or double-membrane vacuoles. Autophagy (2014) 10:1588–602. doi: 10.4161/auto.29411 PMC4206537 25046114

[50] Neal MD Leaphart C Levy R Prince J Billiar TR Watkins S. Enterocyte TLR4 mediates phagocytosis and translocation of bacteria across the intestinal barrier. J Immunol (2006) 176:3070–9. doi: 10.4049/jimmunol.176.5.3070 16493066

[51] Cadwell K. Crosstalk between autophagy and inflammatory signalling pathways: balancing defence and homeostasis. Nat Rev Immunol (2016) 16:661–75. doi: 10.1038/nri.2016.100 PMC5343289 27694913

[52] Sprenkeler EGG Gresnigt MS van de Veerdonk FL. LC3-associated phagocytosis: a crucial mechanism for antifungal host defence against aspergillus fumigatus. Cell Microbiol (2016) 18:1208–16. doi: 10.1111/cmi.12616 27185357

[53] Oikonomou V Renga G De Luca A Borghi M Pariano M Puccetti M. Autophagy and LAP in the fight against fungal infections: Regulation and therapeutics. Mediators Inflammation (2018) 2018:6195958. doi: 10.1155/2018/6195958 PMC5859860 29692681

[54] Bertuzzi M Schrettl M Alcazar-Fuoli L Cairns TC Munoz A Walker LA. The pH-responsive PacC transcription factor of aspergillus fumigatus governs epithelial entry and tissue invasion during pulmonary aspergillosis. PLos Pathog (2014) 10:e1004413. doi: 10.1371/journal.ppat.1004413 25329394 PMC4199764

[55] Nash TJ Morris KM Mabbott NA Vervelde L. Inside-out chicken enteroids with leukocyte component as a model to study host–pathogen interactions. Commun Biol (2021) 4:377. doi: 10.1038/s42003-021-01901-z 33742093 PMC7979936

[56] Schulz O Pabst O. Antigen sampling in the small intestine. Trends Immunol (2013) 34:155–61. doi: 10.1016/j.it.2012.09.006 23083727

[57] Yu LC-H Shih Y-A Wu L-L Lin Y-D Kuo W-T Peng W-H. Enteric dysbiosis promotes antibiotic-resistant bacterial infection: systemic dissemination of resistant and commensal bacteria through epithelial transcytosis. Am J Physiology-Gastrointestinal Liver Physiol (2014) 307:G824–35. doi: 10.1152/ajpgi.00070.2014 PMC4214854 25059827

[58] Chu H Khosravi A Kusumawardhani IP Kwon AH Vasconcelos AC Cunha LD. Gene-microbiota interactions contribute to the pathogenesis of inflammatory bowel disease. Science (2016) 352:1116–20. doi: 10.1126/science.aad9948 PMC4996125 27230380

[59] Rios D Wood MB Li J Chassaing B Gewirtz AT Williams IR. Antigen sampling by intestinal m cells is the principal pathway initiating mucosal IgA production to commensal enteric bacteria. Mucosal Immunol (2016) 9:907–16. doi: 10.1038/mi.2015.121 PMC4917673 26601902

[60] Fasciano AC Blutt SE Estes MK Mecsas J. Induced differentiation of m cell-like cells in human stem cell-derived ileal enteroid monolayers. J Vis Exp (2019) 149:e59894. doi: 10.3791/59894-v PMC6755909 31403623

[61] Staab JF Doucet M Latanich R Lee S Estes MK Kaper JB. Coronin-1 is necessary for enteric pathogen-induced transcytosis across human ileal enteroid monolayers expressing m cells. bioRxiv (2020). 2020.2010.2012.305565. doi: 10.1101/2020.10.12.305565

[62] Hsu NY Nayar S Gettler K Talware S Giri M Alter I. NOX1 is essential for TNFalpha-induced intestinal epithelial ROS secretion and inhibits m cell signatures. Gut (2022). doi: 10.1136/gutjnl-2021-326305 PMC9998338 36191961

[63] Doron I Leonardi I Li XV Fiers WD Semon A Bialt-DeCelie M. Human gut mycobiota tune immunity *via* CARD9-dependent induction of anti-fungal IgG antibodies. Cell (2021) 184:1017–1031.e1014. doi: 10.1016/j.cell.2021.01.016 33548172 PMC7936855

[64] Doron I Mesko M Li XV Kusakabe T Leonardi I Shaw DG. Mycobiota-induced IgA antibodies regulate fungal commensalism in the gut and are dysregulated in crohn's disease. Nat Microbiol (2021) 6:1493–504. doi: 10.1038/s41564-021-00983-z PMC8622360 34811531

[65] Leonardi I Gao IH Lin WY Allen M Li XV Fiers WD. Mucosal fungi promote gut barrier function and social behavior *via* type 17 immunity. Cell (2022) 185:831–846.e814. doi: 10.1016/j.cell.2022.01.017 35176228 PMC8897247

[66] Hershberg RM Mayer LF. Antigen processing and presentation by intestinal epithelial cells - polarity and complexity. Immunol Today (2000) 21:123–8. doi: 10.1016/S0167-5699(99)01575-3 10689299

[67] Rabinowitz K Mayer L. Working out mechanisms of controlled/physiologic inflammation in the GI tract. Immunol Res (2012) 54:14–24. doi: 10.1007/s12026-012-8315-5 22466933 PMC3865894

[68] Beyaz S Chung C Mou H Bauer-Rowe KE Xifaras ME Ergin I. Dietary suppression of MHC class II expression in intestinal epithelial cells enhances intestinal tumorigenesis. Cell Stem Cell (2021) 28:1922–1935.e1925. doi: 10.1016/j.stem.2021.08.007 34529935 PMC8650761

[69] Heuberger C Pott J Maloy KJ. Why do intestinal epithelial cells express MHC class II? Immunology (2021) 162:357–67. doi: 10.1111/imm.13270 PMC7968399 32966619

[70] Koyama M Mukhopadhyay P Schuster IS Henden AS Hulsdunker J Varelias A. MHC class II antigen presentation by the intestinal epithelium initiates graft-versus-Host disease and is influenced by the microbiota. Immunity (2019) 51:885–898.e887. doi: 10.1016/j.immuni.2019.08.011 31542340 PMC6959419

[71] Wosen JE Ilstad-Minnihan A Co JY Jiang W Mukhopadhyay D Fernandez-Becker NQ. Human intestinal enteroids model MHC-II in the gut epithelium. Front Immunol (2019) 10:1970. doi: 10.3389/fimmu.2019.01970 31481960 PMC6710476

[72] Iliev ID Cadwell K. Effects of intestinal fungi and viruses on immune responses and inflammatory bowel diseases. Gastroenterology (2021) 160:1050–66. doi: 10.1053/j.gastro.2020.06.100 PMC7956156 33347881

[73] Fujii M Sato T. Somatic cell-derived organoids as prototypes of human epithelial tissues and diseases. Nat Materials (2021) 20:156–69. doi: 10.1038/s41563-020-0754-0 32807924

[74] Lee IH Cao L Mostoslavsky R Lombard DB Liu J Bruns NE. A role for the NAD-dependent deacetylase Sirt1 in the regulation of autophagy. Proc Natl Acad Sci USA (2008) 105:3374–9. doi: 10.1073/pnas.0712145105 PMC2265142 18296641

[75] Sato T Stange DE Ferrante M Vries RG Van Es JH Van den Brink S. Long-term expansion of epithelial organoids from human colon, adenoma, adenocarcinoma, and Barrett's epithelium. Gastroenterology (2011) 141(5):1762–72. doi: 10.1053/j.gastro.2011.07.050 21889923

[76] Usui T Sakurai M Umata K Yamawaki H Ohama T Sato K. Preparation of human primary colon tissue-derived organoid using air liquid interface culture. Curr Protoc Toxicol (2018) 75:22 26 21–22 26 27. doi: 10.1002/cptx.40 29512123

[77] Pleguezuelos-Manzano C Puschhof J van den Brink S Geurts V Beumer J Clevers H. Establishment and culture of human intestinal organoids derived from adult stem cells. Curr Protoc Immunol (2020) 130:e106. doi: 10.1002/cpim.106 32940424 PMC9285512

[78] Wheeler RT Fink GR. A drug-sensitive genetic network masks fungi from the immune system. PLos Pathog (2006) 2:e35. doi: 10.1371/journal.ppat.0020035 16652171 PMC1447670

[79] Marakalala MJ. *In vitro* analysis for macrophage binding and pro-inflammatory responses to candida albicans. Bio Protoc 4 (2014) 4(9):e1123. doi: 10.21769/BioProtoc.1123 PMC5718290 29226178

