# Supplementary material for: Human intestinal epithelial cells can internalize luminal fungi via LC3-associated phagocytosis
Source: Front Immunol. 2023 Mar 8;14:1142492. doi: 10.3389/fimmu.2023.1142492 (PMC10030769; doi:10.3389/fimmu.2023.1142492)
Supplement: Supplementary file 3 [file DataSheet_1.pdf]

Supplementary Material

Human intestinal epithelial cells can internalize luminal fungi via LC3-associated phagocytosis

Sarit Cohen-Kedar, Efrat Shaham Barda, Keren Masha Rabinowitz, Danielle Keizer, Hanan Abu-Taha, Shoshana Schwartz, Kawsar Kaboub, Liran Baram, Eran Sadot, Ian White, Nir Wasserberg, Meirav Wolff-Bar, Adva Levy-Barda and Iris Dotan

Corresponding authors: Iris Dotan and Sarit Cohen-Kedar: irisdo@clalit.org.il, sarit.cohenkedar@gmail.com

Supplementary Methods

Fluorescent immunohistochemistry of frozen sections

Mucosal samples were taken from surgical specimens of patients undergoing bowel resection for colonic tumors. Normal mucosa was taken from a distance of at least 10 cm from the tumor and was snap-frozen in Tissue–Plus OCT compound (Scigen scientific Gardena) and kept at -80°C until sectioning for immunofluorescence.

Frozen sections (10 µM) were stained using a standard protocol according to the procedure in "Immunofluorescence" in the main text.

Isolation of primary human IECs for flow cytometry

IECs were freshly isolated as previously described (Brimnes et al., 2005; Rimoldi et al., 2005) with modifications. Briefly, mucosa and submucosa were separated. The tissue was dissected and incubated in 30 mL of HBSS without Ca2+ and Mg2+, containing 1 mM dithiothreitol, 5% FBS and 2 mM EDTA for 30 min at 37°C, while agitating at 250 rpm. The suspension was filtered through nylon mesh (70 mm pore diameter) and the pellet containing IECs was used flow cytometry.

Flow cytometry

Cell surface immunostaining of IECs was performed using APC-conjugated anti-human Dectin-2 mAb (clone 545943, FAB3114, R&D systems) according to the manufacturer's instructions. All studies were carried out using matched isotype controls. Primary IECs were identified by co-staining with APC-conjugated human EpCAM mAb (clone 9C4,

324208, Biolegend). Data acquisition was performed using a FACSCanto flow cytometer (Becton Dickinson) and the data were analyzed with FlowJo software (Treestar, Inc.).

Supplementary References

Brimnes, J., Allez, M., Dotan, I., Shao, L., Nakazawa, A., and Mayer, L. (2005). Defects in CD8+ regulatory T cells in the lamina propria of patients with inflammatory bowel disease. J Immunol *174*, 5814-5822.

Rimoldi, M., Chieppa, M., Salucci, V., Avogadri, F., Sonzogni, A., Sampietro, G.M., Nespoli, A., Viale, G., Allavena, P., and Rescigno, M. (2005). Intestinal immune homeostasis is regulated by the crosstalk between epithelial cells and dendritic cells. Nat Immunol *6*, 507-514.

Supplementary figure legends

Supp. Fig. S1: HCT116 cells uptake of zymosan depends on actin polymerization and Dectin-1.

(A) HCT116 were fed with pHrodo-red zymosan overnight and stained with Hoechst 33342 prior to live imaging. Arrowhead – intracellular fluorescent zymosan, arrow-intracellular fragmented zymosan. Original magnification x20, scale bar 10 µm.

(B) HCT116 were fed with pHrodo-red zymosan overnight in the presence or absence of Cytochalasin D (10 µM) or laminarin (1 mg/ml) which were added 1 hour prior to zymosan, and stained with Hoechst 33342 before live imaging. Original magnification x20, scale bar 50 µm. (C,D) HCT116 cells were seeded in 96 well plate and treated as in (B) and phagocytosis was assessed as the relative fluorescence by a microplate reader. Data are shown as mean ± SD of biological triplicates from a representative of three independent experiments performed. ***$p \leq 0.001$, Unpaired *t-test* vs. no inhibitor.

Supp Fig. S2: Caco-2 cells internalize and process zymosan. (A) Caco-2 cells were seeded on glass slides and fed with pHrodo-red zymosan overnight. Following fixation cells were immuno- stained with zo-1 to mark cell borderline (green) and nuclei were counterstained with DAPI (blue). Multiple intracellular zymosan particles are shown (white arrowheads) as well as extracellular zymosan (black arrowhead). Original magnification x20, scale bar 20 µm. (B) Live imaging of Caco-2 cells fed with pHrodo-red

zymosan overnight shows fluorescent internalize zymosan (red). Intact (arrowheads) and fragmented processed (arrow) zymosan are indicated. Original magnification x40, scale bar 20 µm. All images are representative of at least three independent experiments performed.

Supp Fig. S3: Wider fields of the images shown in Fig. 1 B-C. Zymosan uptake is sensitive to cytochalasin-D. SW480 cells were seeded on glass-bottom chambers as indicated in Methods, and fed overnight with pHrodo-red zymosan in the absence (A) or presence (B) of cytochalasin-D (10 µM) and counter stained with Hoechst 33342 (blue) prior to confocal live imaging. Original magnification x20, scale bar 10 µm.

Supp Fig. S4: Phagocytosis of pHrodo-red zymosan by ileal organoids. Ileal organoids were grown as monolayers in expansion medium and let do differentiate for 2 days. PHrodo-red zymosan was added to the medium for 24h, and intracellular zymosan became red. Original magnification x10, scale bar 200 µm. Shown are representative images (from which a selected frame is shown in figure 2A), from 3-5 randomly acquired scans of 4 ileal and organoids.

Supp Fig. S5: Phagocytosis of pHrodo-red zymosan by colonic organoids. Colonic organoids were grown as monolayers in expansion medium and let do differentiate for 2 days (A), or left in expansion medium (B). PHrodo-red zymosan (red) was added to the medium for 24h. Original magnification x10, scale bar 50 µm. Shown are representative images (from which selected frames (while squares) are enlarged in the insets), from 3-5 randomly acquired scans of two independent experiments of two organoids.

Supp Fig. S6: Identification of epithelial subtypes in ileal organoid monolayers. Organoids from human terminal ilium were grown in monolayers in “expansion medium” and then the medium was replaced to differentiation medium for 3 days. Following PFA fixation and permeabilization, organoids were stained with (A) Muc2 (green) and Ki-67 (red) or (B) lysozyme (Magenta) and DAPI (blue). Original magnification x20, scale bars 100 µm (A), 20 µm (B). Arrows indicate lysosome granules.

Supp Fig. S7: Identification of epithelial subtypes in colonic organoid monolayers. Organoids from human colon were grown in monolayers in “expansion medium” and

then the medium was replaced to differentiation medium for 3 days. Following PFA fixation and permeabilization, organoids were stained with (A) MUC2 (green) and (B) Ki-67 (red) and DAPI (blue). Original magnification x20, scale bars 100 µm

Supp Fig. S8: Zymosan is phagocytosed by goblet and non-goblet cells. Colonic organoids were fed overnight with pHrodo red zymosan (red), fixed and stained with MUC2 antibody (green) and DAPI (blue). Both goblet (MUC2+, arrowheads) and non-goblet (MUC2-, arrows) cells phagocytosed zymosan. Magnification x20, scale bar 20 µm.

Supp Fig. S9: Dectin-1 is expressed mostly at the distal-peripheral region of colonic organoids. Colonic organoids were grown in monolayer and let to differentiate for 3 days. Following fixation with PFA, organoids were stained with Dectin-1 monoclonal antibody (magenta) and EpCAM polyclonal antibody (orange) and counterstained with DAPI (blue). While EpCAM staining is uniform in most of the organoid, Dectin-1 is enhanced mostly at the periphery of the organoid (arrow head), and less to the internal parts (arrows). Original magnification x20, scale bar 50 µm.

Supp Fig. S10: Dectin-1 is expressed in the luminal face of human intestinal crypts.
(A, B) Frozen sections of human colon surgical specimens were stained with Dectin-1 polyclonal antibody (red) and EpCAM monoclonal antibody (green) and counterstained with DAPI (A, cyan). (A) shows that Dectin-1 is enhanced in the lumen facing IECs (arrowheads) while EpCAM stains uniformly IECs along the crypt, and (B) demonstrates that Dectin-1 is located to the apical face IECs (arrowheads). Original magnification x20, scale bar 100 µm(A) and 20 µm (B).

Supp Fig. S11: Dectin-1 is recruited to internalized zymosan.
Colonic organoids were fed with pHrodo red zymosan and stained with FITC labeled Dectin-1 monoclonal antibody. Original magnification x63, scale bar 5µm.

Supp Fig. S12: Phagocytosis of *C. albicans* by IECs. (A) SW480 cells were fed overnight with Rhodamine-green labeled HK *C. albicans*. Live images were taken (z-stack 1 µm step). Arrow- intracellular fragmented *C. albicans*. (B) Ileal organoids were fed overnight with Rhodamine-green labeled UV-inactivated *C. albicans*, and stained with Hoechst

33342 prior to live confocal imaging. Arrow- intracellular fragmented *C. albicans*. Original magnification x20, scale bar 10 µm (A) 20 µm (B).

Supp Fig. S13: *C. albicans* is phagocytosed by goblet and non-goblet cells. Colonic organoids grown as monolayers were fed overnight with rhodamine-green labelled HK-*C. albicans* (green), fixed and stained with MUC2 antibody (red) and DAPI (blue). Both goblet ($MUC2^{+}$, arrowheads) and non-goblet ($MUC2^{-}$, arrows) cells phagocytosed *C. albicans*. Fragmented and diffused *C. albicans* (green arrows) indicate intracellular localization and processing. Magnification x20, scale bar 50 µm.

Supp Fig. S14: IECs can phagocytose both zymosan and *C. albicans*. Ileal (A) and colonic (B) organoids were fed overnight with pHrodo-red zymosan and Rhodamine-green-X labeled HK- *C. albicans* and stained with Hoechst 33342 prior to confocal live imaging. Arrows indicate cells that phagocytosed both *zymosan and C. albicans.* Original magnification x10 (A) x40 (B), scale bar 100 µm (A) and 20 µm (B).

Supp Fig. S15: Dectin-2 is expressed on primary human IECs. (A) EpCAM-based flow cytometric gating on IECs. Freshly isolated human IEC-enriched preparation was stained with EpCAM and CD45 antibodies to identify IECs and leukocytes respectively. (B-C) Human IEC cell surface expression of Dectin-2. IECs generated from the (B) ileum and (C) colon of the same donor were stained as above, as well as with Dectin-2 Ab. Dectin-2 expression by intestinal epithelial cells (gated EpCAM+ population) was assessed by flow cytometry (filled gray histograms) and was compared with isotype-matched control Ab (open histograms). The data shown are representative of at least 3 individuals.

(D) Frozen sections of human colon surgical specimen were stained with Dectin-2 monoclonal antibody (green) and EpCAM polyclonal antibody (red) and counterstained with DAPI (blue). Original magnification x20, scale bar 50 µm. The images are representative of three individuals tested.

Supp Fig. S16: LC3 is recruited to phagocytosed particles.

SW480 cells (A) and Ileal organoids (B) were fed with Rhodamine-green-X HK- *C. albicans* and stained with LC3 (magenta) and DAPI (blue). Original magnification x20 (A) x63 (B), scale bar 10 µm.

Supp Fig. S17: Phagocytosed particles are directed to the lysosomes.
(A, B) Colonic organoids were fed with UV-inactivated *C. albicans* (green) and stained with lysosomal-NIR reagent (Magenta). Processed (A, arrow) and intact (A, B, arrowheads) particles are indicated, and co-localized with lysosomes. Original magnification x40 (A) x63 (B), scale bar 10 µm
C) HCT116 cells were fed with AF594-zymosan (red) and stained with LAMP2 antibody (green) and DAPI (blue). Original magnification x63, scale bar 10 µm

Supp Movie 1: Zymosan uptake by intestinal organoids (related to Fig. 2C). differentiated monolayers of colonic organoids were fed with pHrodo-red zymosan for 24 hours. nuclei were stained with Hoechst 33342 (blue) prior to confocal live imaging. Shown a movie of the sequential frames acquired by z-stack analysis. Original magnification x63, scale bar 5 µm.
Supp. Movie 2: Intestinal organoids uptake zymosan (related to Fig. 2C). Colonic organoids were treated as in Supp. Movie 1. Shown a movie of the sequential frames acquired by z-stack analysis. Original magnification x63, scale bar 10 µm.
