# Supplementary material for: Human intestinal epithelial cells can internalize luminal fungi via LC3-associated phagocytosis
Source: Front Immunol. 2023 Mar 8;14:1142492. doi: 10.3389/fimmu.2023.1142492 (PMC10030769; doi:10.3389/fimmu.2023.1142492)
Supplement: Supplementary file 4 [file DataSheet_2.pdf]

Supp. Fig. S1

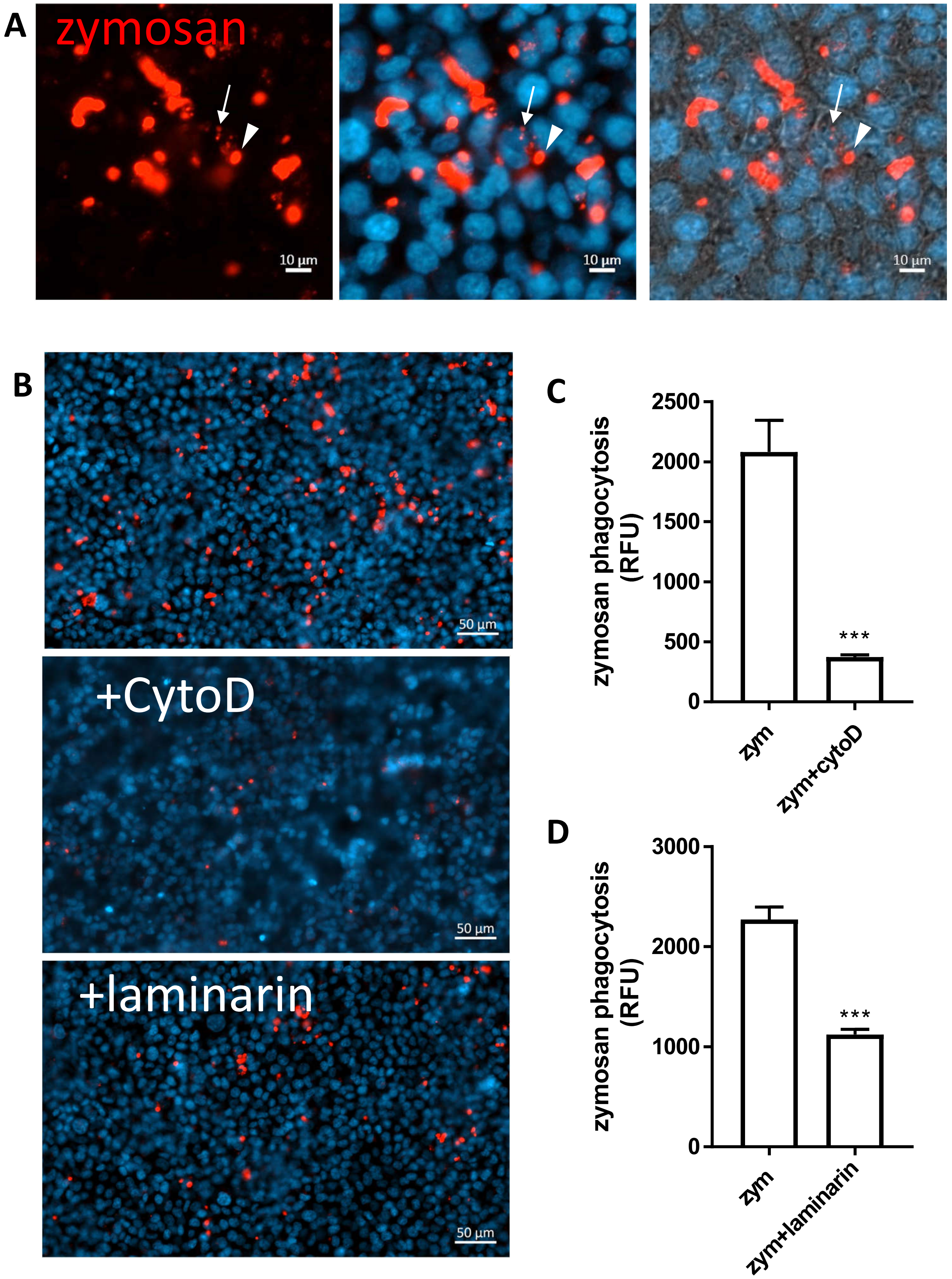
A
zymosan
10 µm
B
+CytoD
+laminarin
50 µm
C
zymosan phagocytosis (RFU)
2500
2000
1500
1000
500
0
***
zym
zym+cytoD
D
zymosan phagocytosis (RFU)
3000
2000
1000
0
***
zym
zym+laminarin

Supp Fig. S1: HCT116 cells uptake of zymosan depends on actin polymerization and Dectin-1.

(A) HCT116 were fed with phrodo-red zymosan overnight and stained with Hoechst 33342 (blue) prior to live imaging. Red Fluorescence indicates intracellular zymosan. Arrowhead – intracellular fluorescent zymosan, arrow- intracellular fragmented zymosan. Original magnification x20, scale bar 10 µm.

(B) HCT116 were fed with phrodo-red zymosan overnight in the presence or absence of Cytochalasin D (10 µM) or laminarin (1 mg/ml) which were added 1 hour prior to zymosan, and stained with Hoechst 33342 before live imaging. Original magnification x20, scale bar 50 µm.

(C,D) HCT116 cells were seeded in 96 well plate and treated as in (B) and phagocytosis was assessed as the relative fluorescence by a plate reader. Data are shown as mean ± SD of biological triplicates from a representative of three independent experiments performed. ***p≤0.001, Unpaired *t-test* vs. no inhibitor.

Supp. Fig. S2

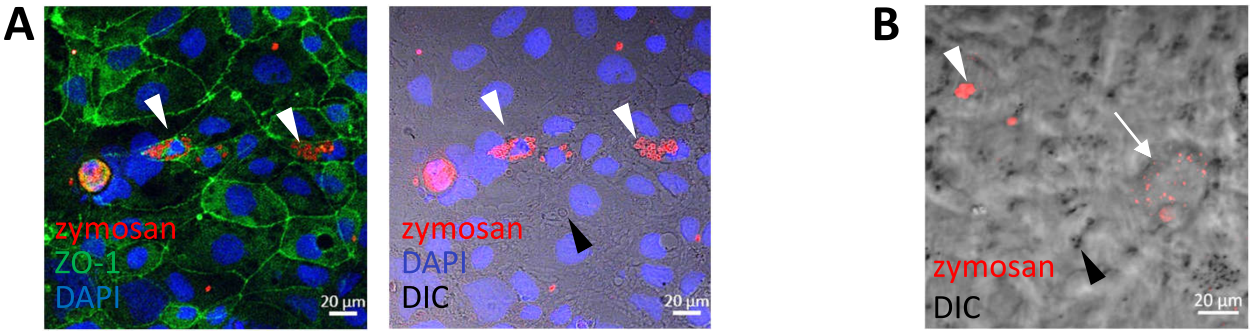

Supp Fig. S2: Caco-2 cells internalize and process zymosan. (A) Caco-2 cells were seeded on glass slides and fed with pHrodo-red zymosan overnight. Following fixation cells were immuno- stained with zo-1 to mark cell borderline (green) and nuclei were counterstained with DAPI (blue). Multiple intracellular zymosan particles are shown (white arrowheads) as well as extracellular zymosan (black arrowhead). Original magnification x20, scale bar 20 µm. (B) Live imaging of Caco-2 cells fed with pHrodo-red zymosan overnight shows fluorescent internalize zymosan (red). Intact (arrowheads) and fragmented processed (arrow) zymosan are indicated. Original magnification x40, scale bar 20 µm. All images are representative of at least three independent experiments performed.

Supp. Fig. S3

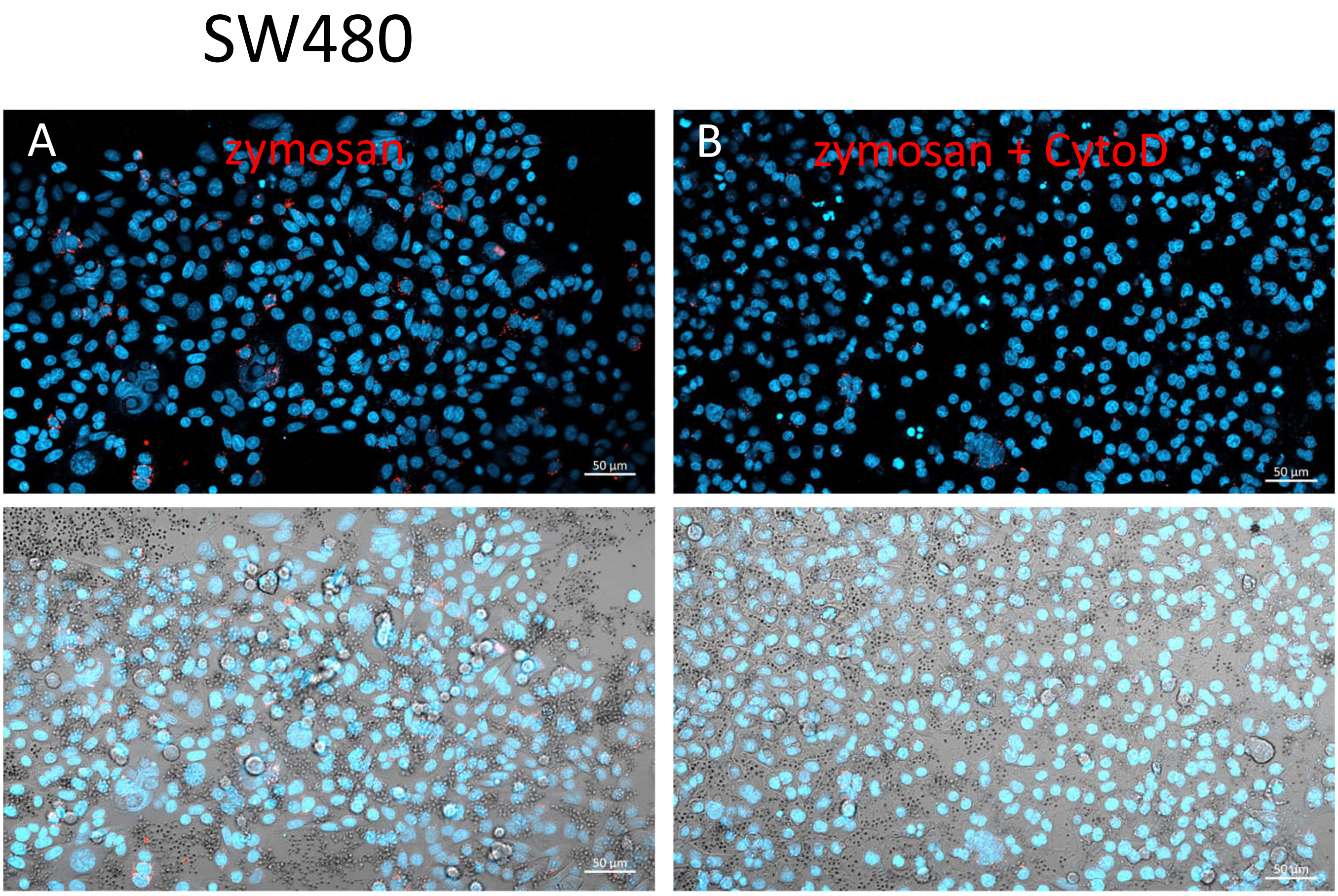

Supp Fig. S3: Wider fields of the images shown in Fig. 1 B-C. Zymosan uptake is sensitive to cytochalasin-D. SW480 cells were seeded on glass-bottom chambers as indicated in Methods, and fed overnight with pHrodo-red zymosan in the absence (A) or presence (B) of cytochalasin-D (10 µM) and counter stained with Hoechst 33342 (blue) prior to confocal live imaging. Original magnification x20, scale bar 10 µm.

Supp. Figure S4

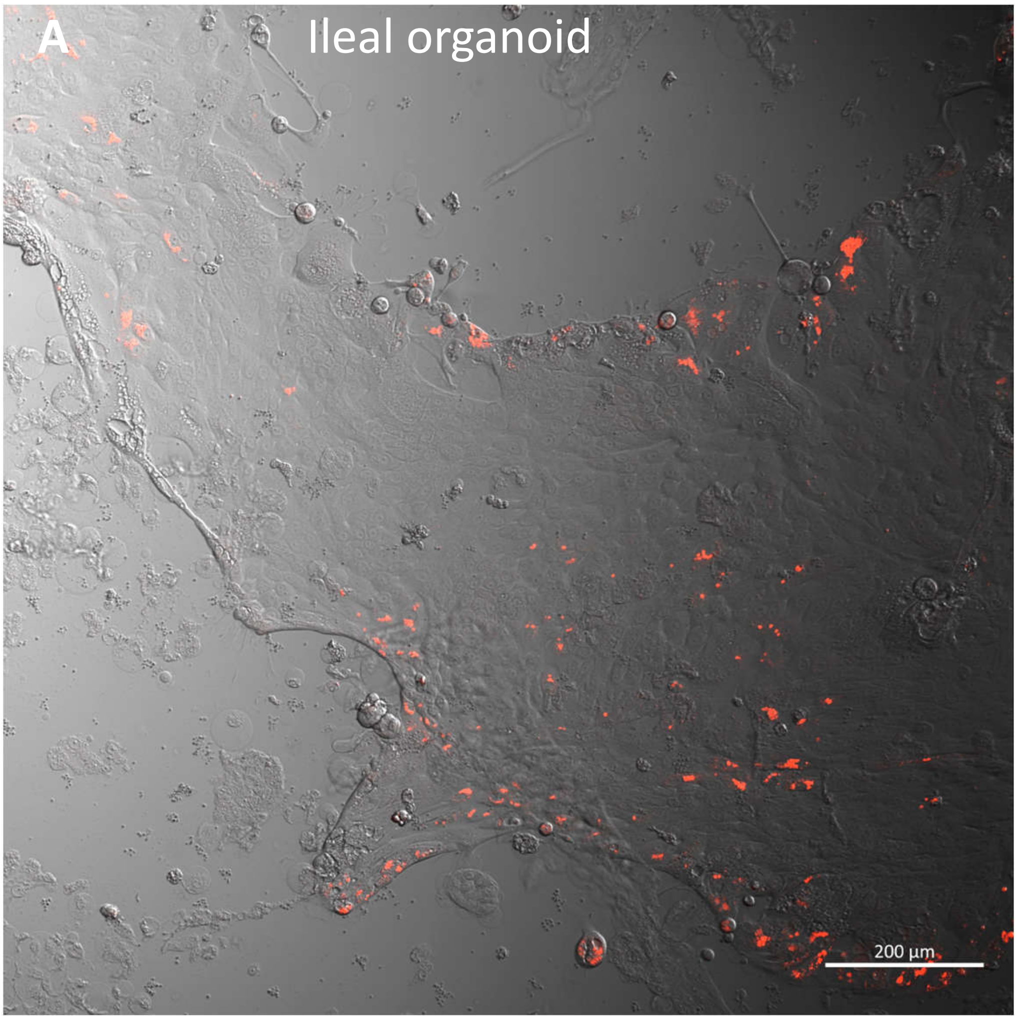

Supp Fig. S4: Phagocytosis of pHrodo-red zymosan by ileal organoids. Ileal organoids were grown as monolayers in expansion medium and let do differentiate for 2 days. PHrodo-red zymosan was added to the medium for 24h, and intracellular zymosan became red. Original magnification x10, scale bar 200 µm. Shown are representative images (from which a selected frame is shown in **Figure 2A**), from 3-5 randomly acquired scans of 4 ileal and organoids.

Supp. Figure S5

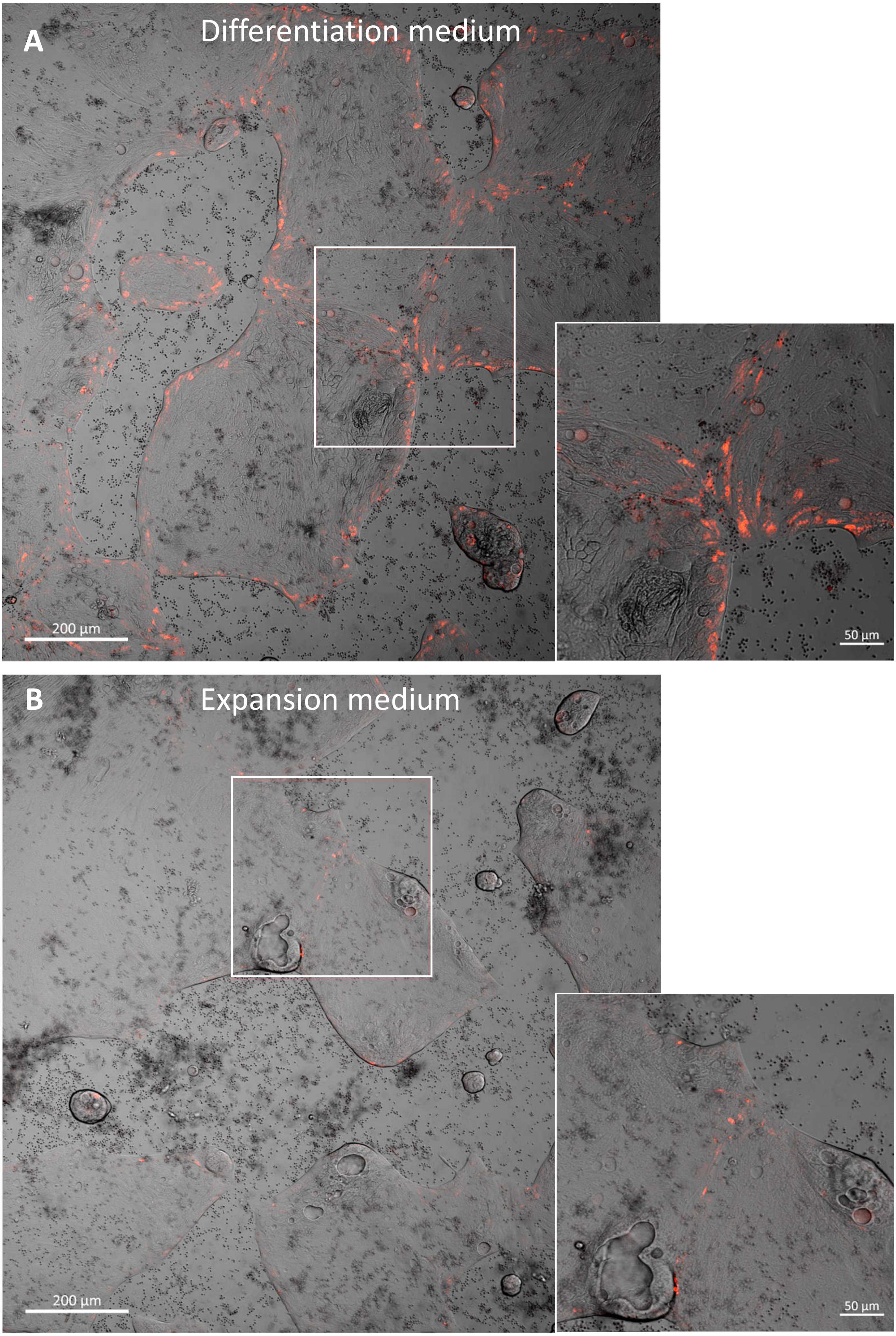

Supp Fig. S5: Phagocytosis of pHrodo-red zymosan by colonic organoids. Colonic organoids were grown as monolayers in expansion medium and let do differentiate for 2 days (A), or left in expansion medium (B). PHrodo-red zymosan (red) was added to the medium for 24h. Original magnification x10, scale bar 50 µm. Shown are representative images (from which selected frames (while squares) are enlarged in the insets), from 3-5 randomly acquired scans of two independent experiments of two organoids.

Supp. Figure S6

A

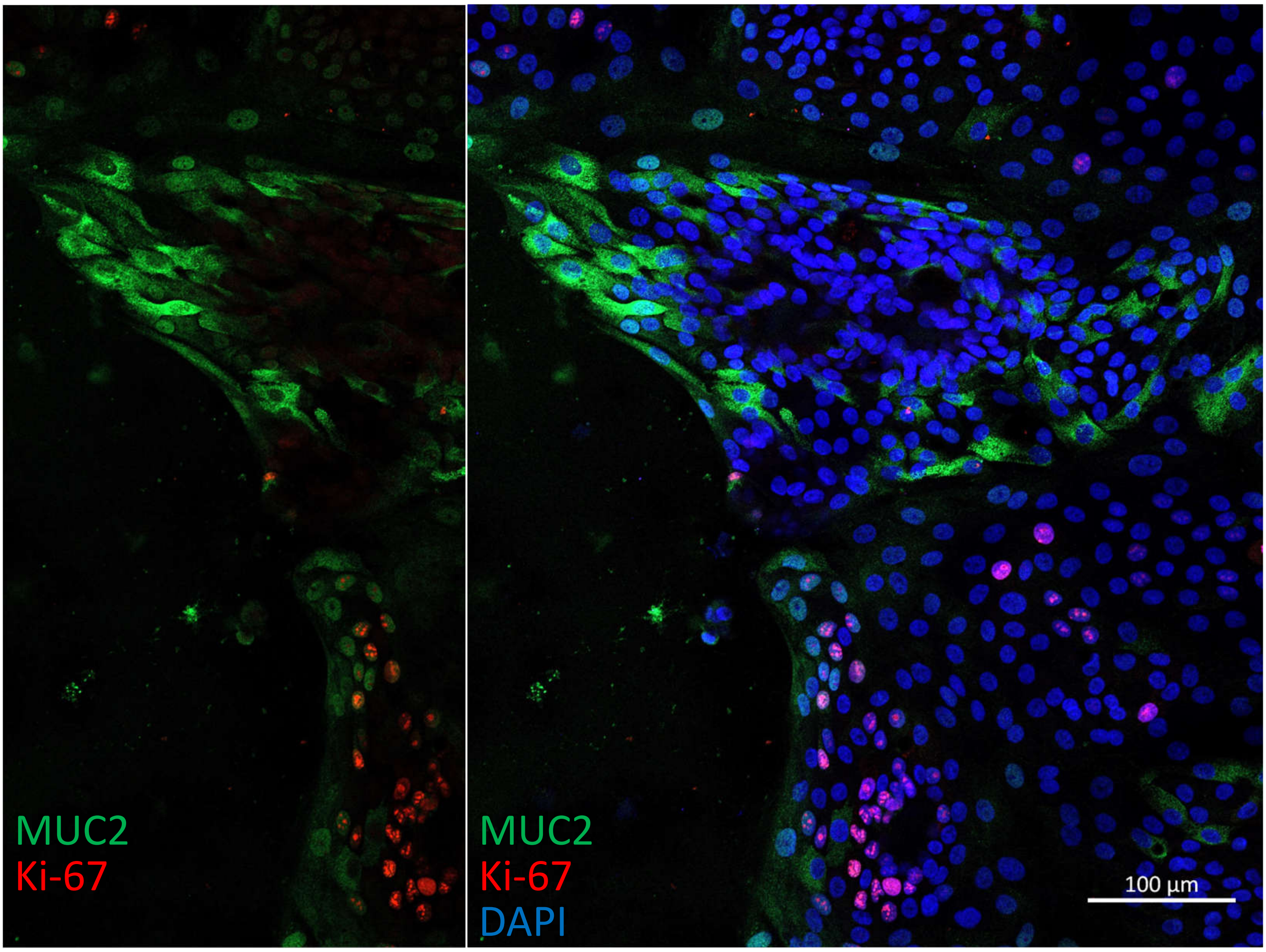

B

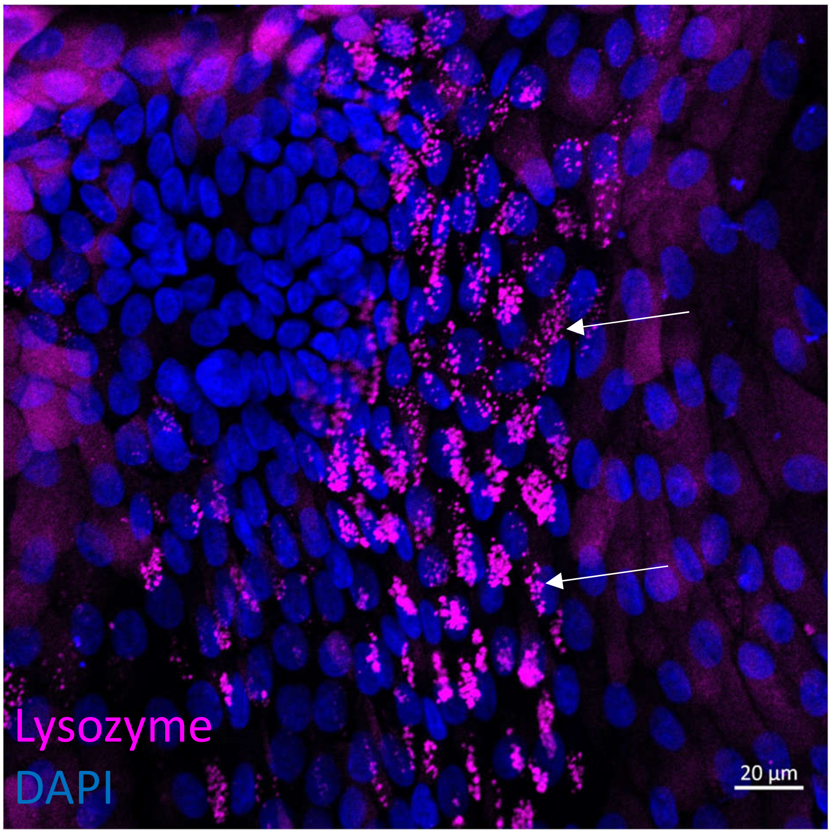

Supp Fig. S6: Identification of epithelial subtypes in ileal organoid monolayers. Organoids from human terminal ilium were grown in monolayers in "expansion medium" and then the medium was replaced to differentiation medium for 3 days. Following PFA fixation and permeabilization, organoids were stained with (A) MUC2 (green) and Ki-67 (red) or (B) lysozyme (Magenta) and DAPI (blue). Original magnification x20, scale bars 100 µm (A), 20 µm (B). Arrows indicate lysosome granules.

Supp. Figure S7

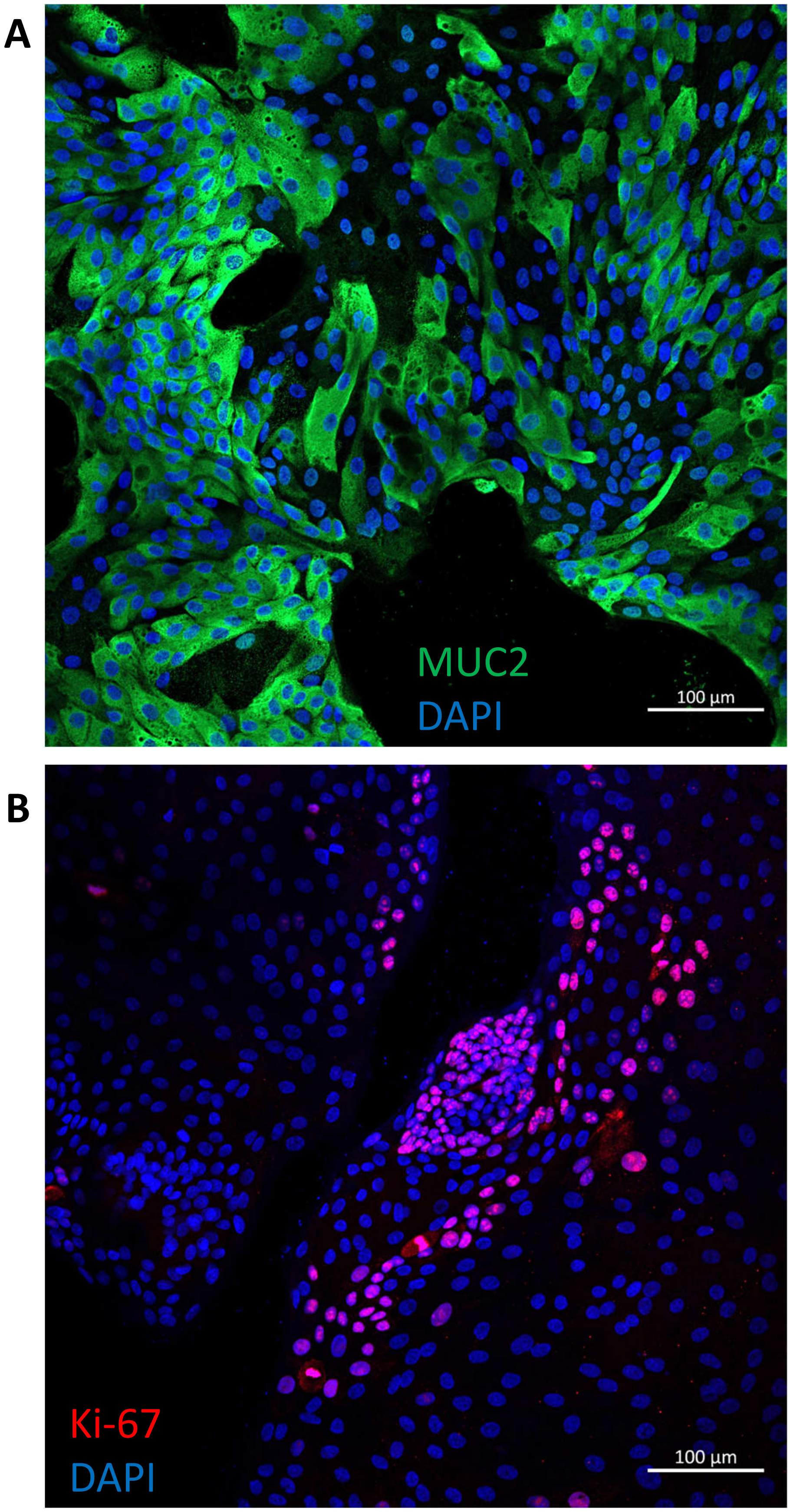

Supp Fig. S7: Identification of epithelial subtypes in colonic organoid monolayers. Organoids from human colon were grown in monolayers in "expansion medium" and then the medium was replaced to differentiation medium for 3 days. Following PFA fixation and permeabilization, organoids were stained with (A) MUC2 (green) and (B) Ki-67 (red) and DAPI (blue). Original magnification x20, scale bars 100 µm

Supp. Figure S8

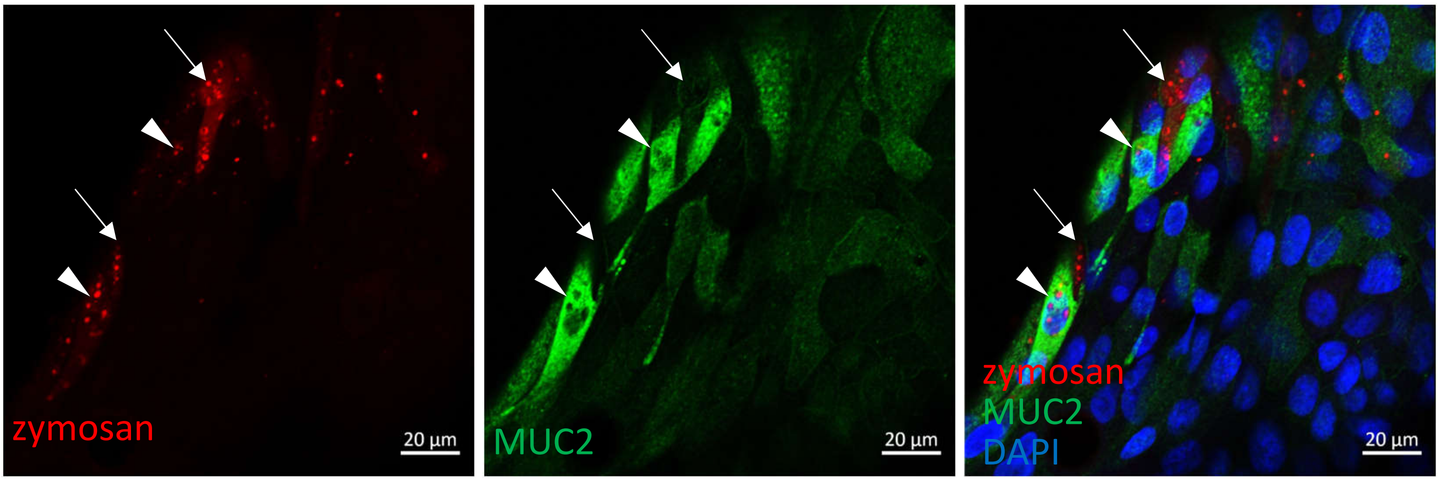

Supp Fig. S8: Zymosan is phagocytosed by goblet and non-goblet cells. Colonic organoids were fed overnight with pHrodo red zymosan (red), fixed and stained with MUC2 antibody (green) and DAPI (blue). Both goblet ($MUC2^+$, arrowheads) and non-goblet ($MUC2^-$, arrows) cells phagocytosed zymosan. Magnification x20, scale bar 20 µm.

Supp. Figure S9

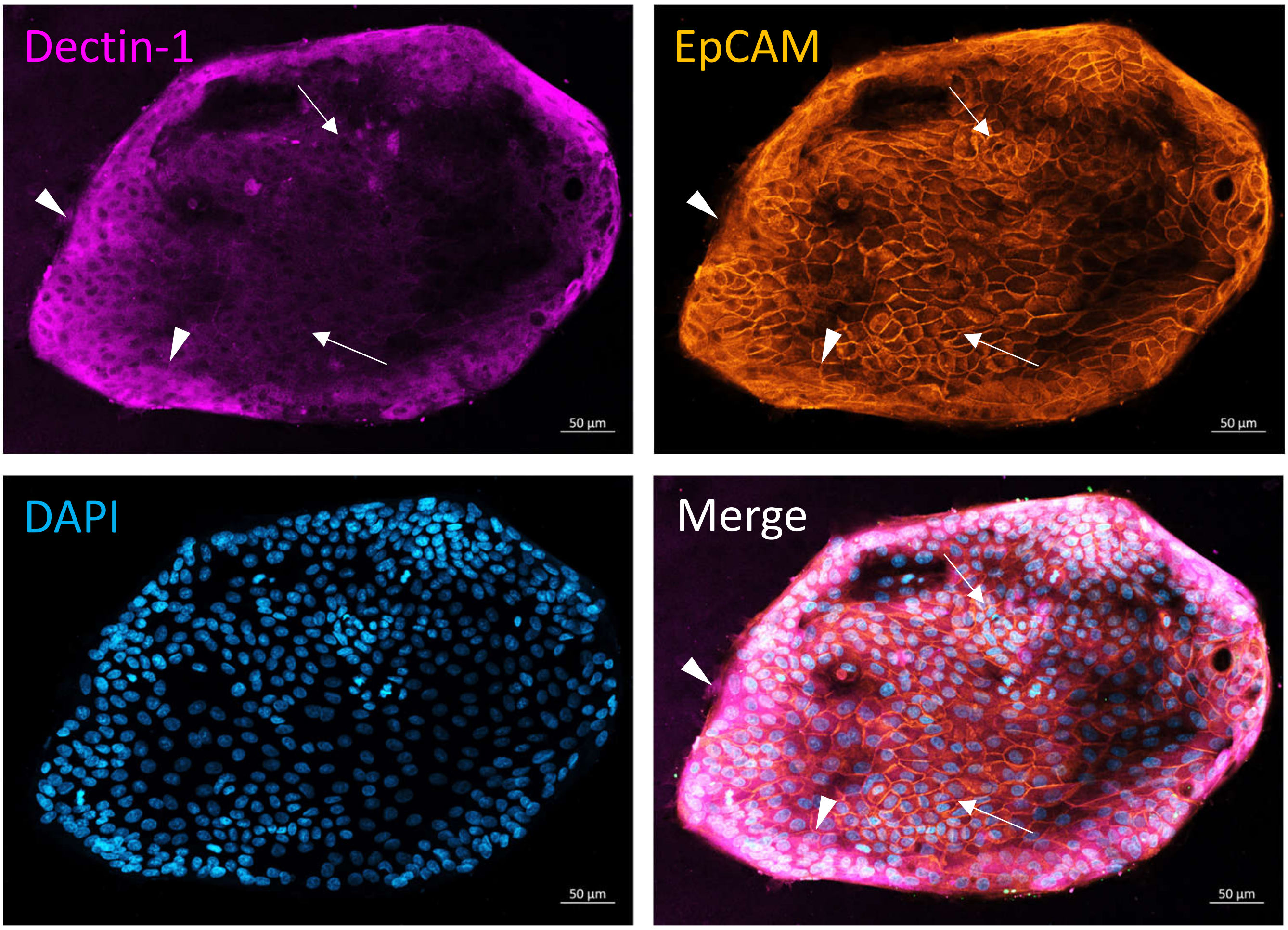

Supp Fig. S9: Dectin-1 is expressed mostly at the distal-peripheral region of colonic organoids. Colonic organoids were grown in monolayer and let to differentiate for 3 days. Following fixation with PFA, organoids were stained with Dectin-1 monoclonal antibody (magenta) and EpCAM polyclonal antibody (orange) and counterstained with DAPI (blue). While EpCAM staining is uniform in most of the organoid, Dectin-1 is enhanced mostly at the periphery of the organoid (arrow head), and less to the internal parts (arrows). Original magnification x20, scale bar 50 µm.

Supp. Figure S10

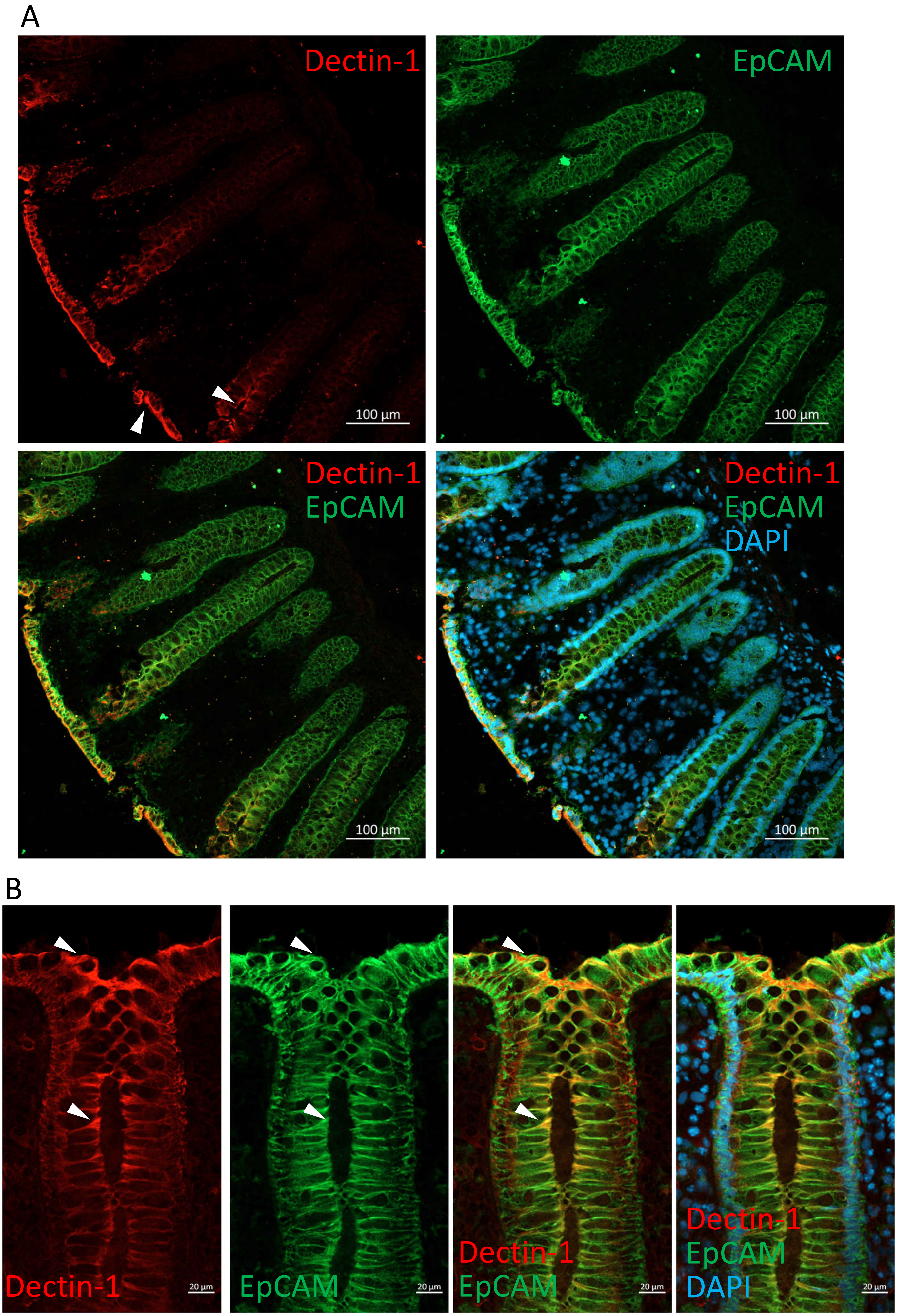

Supp Fig. S10: Dectin-1 is expressed in the luminal face of human intestinal crypts. (A, B) Frozen sections of human colon surgical specimen were stained with Dectin-1 polyclonal antibody (red) and EpCAM monoclonal antibody (green) and counterstained with DAPI (A, cyan). (A) shows that Dectin-1 is enhanced in the lumen facing IECs (arrowheads) while EpCAM stains uniformly IECs along the crypt, and (B) demonstrates that Dectin-1 is located to the apical face IECs (arrowheads). Original magnification x20, scale bar 100 µm(A) and 20 µm (B).

Supp. Figure S11

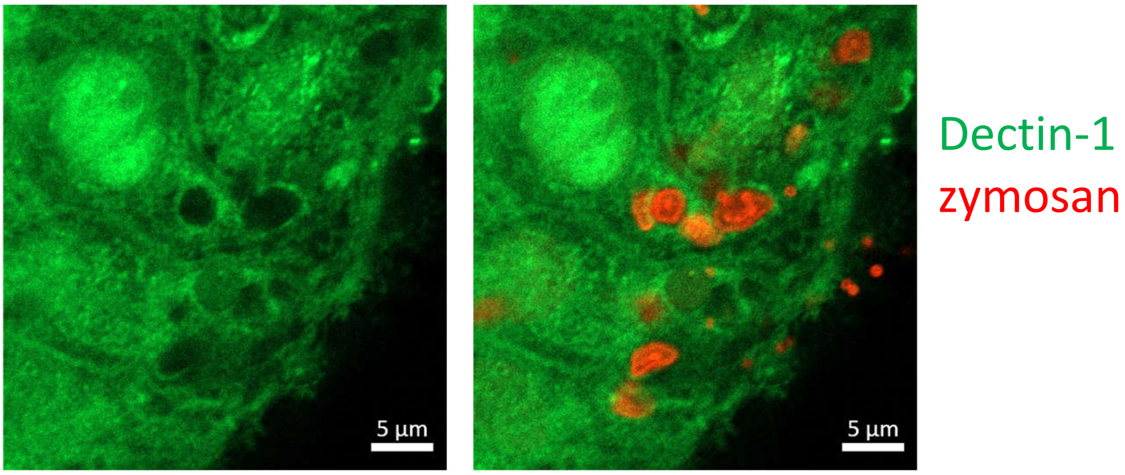

Supp Fig. S11: Dectin-1 is recruited to internalized zymosan.
Colonic organoids were fed with pHrodo red zymosan and stained with FITC labeled Dectin-1 monoclonal antibody. Original magnification x63, scale bar 5µm.

Supp. S12

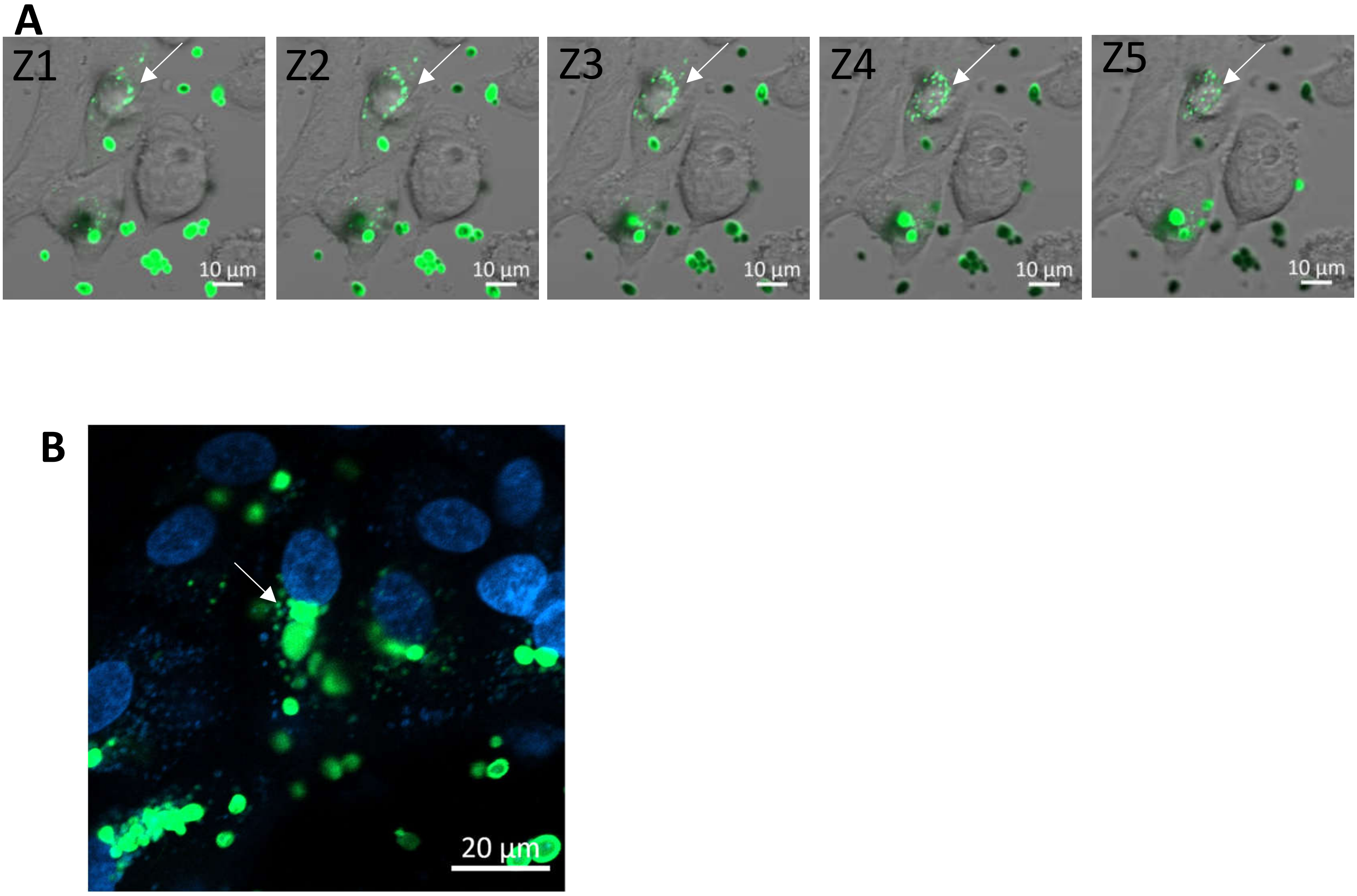

Supp Fig. S12: Phagocytosis of *C. albicans* by IECs. (A) SW480 cells were fed overnight with Rhodamine-green labeled HK *C. albicans*. Live images were taken (z-stack 1 µm step). Arrow- intracellular fragmented *C. albicans*. (B) Ileal organoids were fed overnight with Rhodamine-green labeled UV-inactivated *C. albicans*, and stained with Hoechst 33342 prior to live confocal imaging. Arrow- intracellular fragmented *C. albicans*. Original magnification x20, scale bar 10 µm (A) 20 µm (B).

Supp. S13

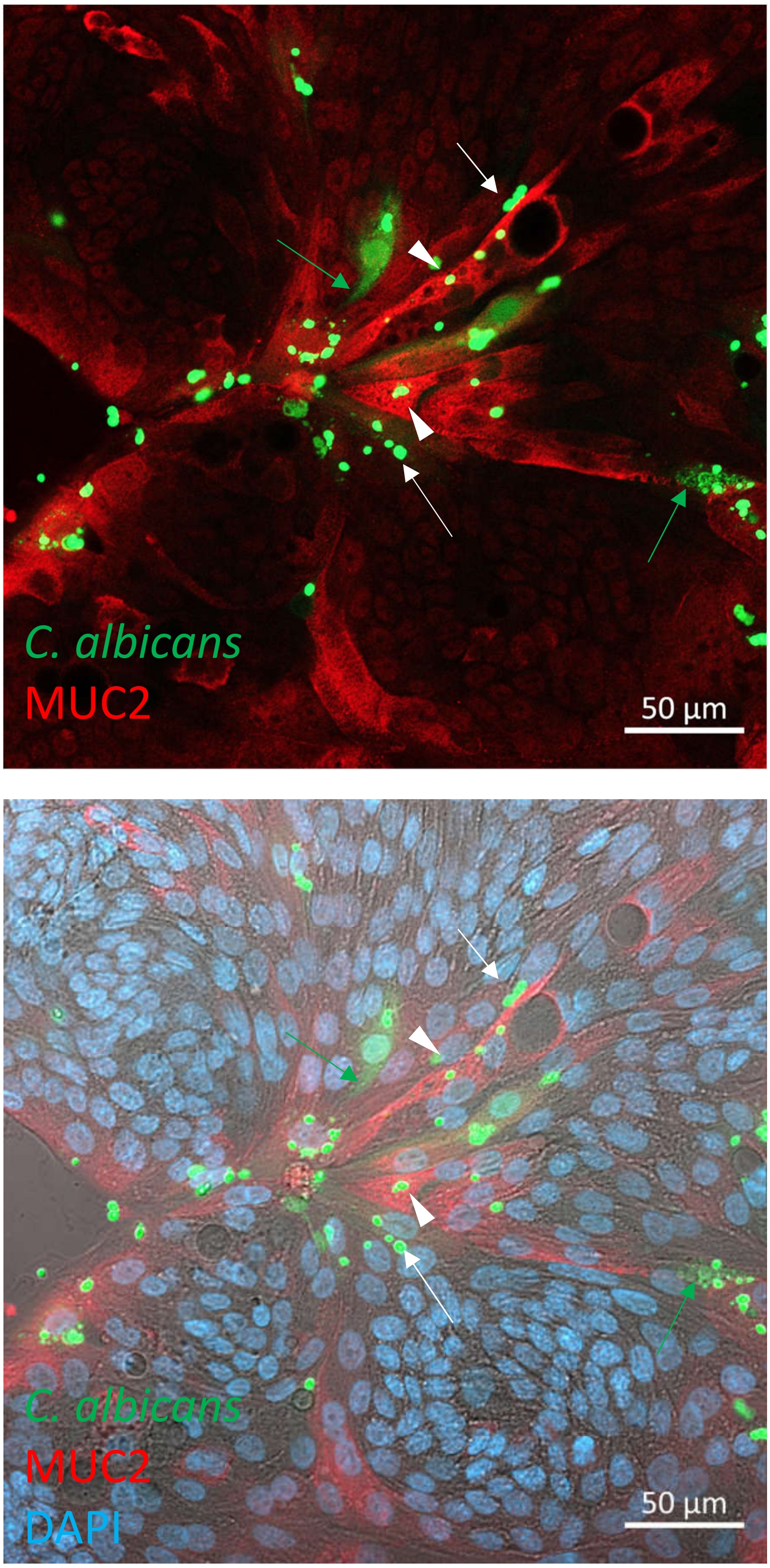

Supp Fig. S13: *C. albicans* is phagocytosed by goblet and non-goblet cells. Colonic organoids grown as monolayers were fed overnight with rhodamine-green labelled HK-*C. albicans* (green), fixed and stained with MUC2 antibody (red) and DAPI (blue). Both goblet ($MUC2^+$, arrowheads) and non-goblet ($MUC2^-$, arrows) cells phagocytosed *C. albicans*. Fragmented and diffused *C. albicans* (green arrows) indicate intracellular localization and processing. Magnification x20, scale bar 50 µm.

Supp. S14

A

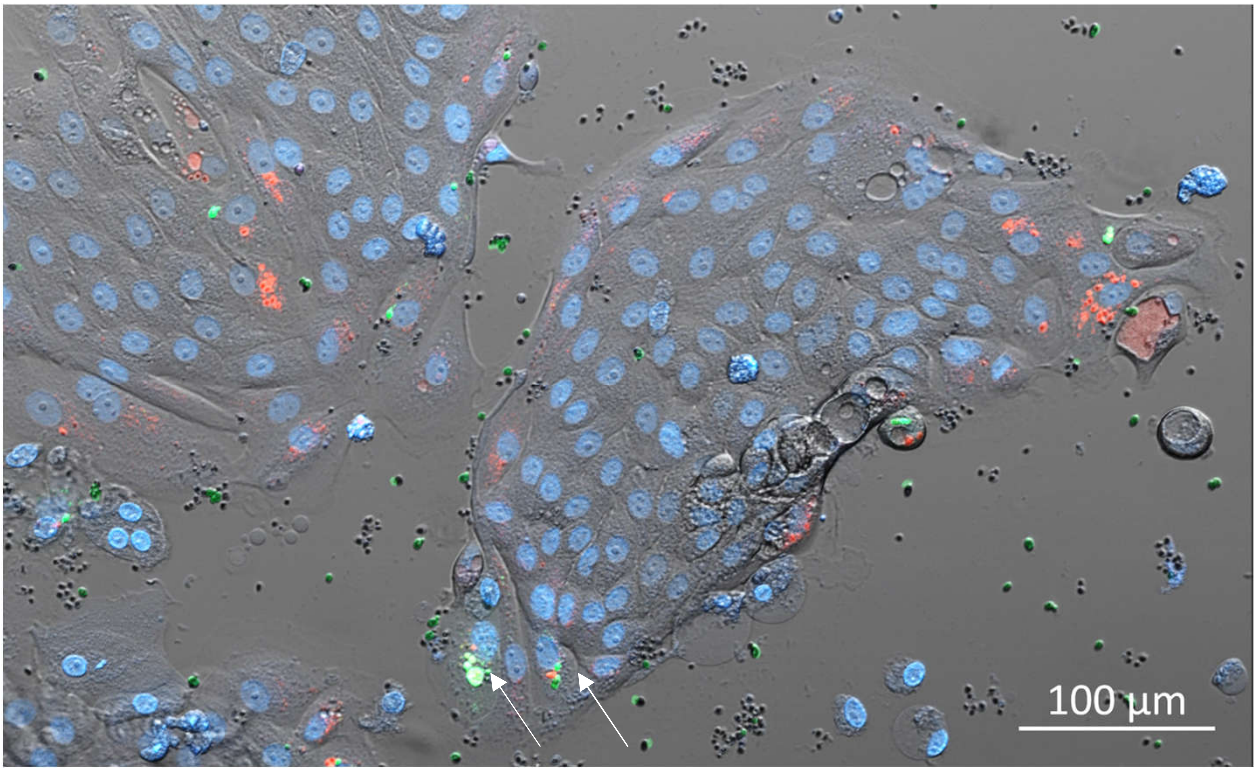

B

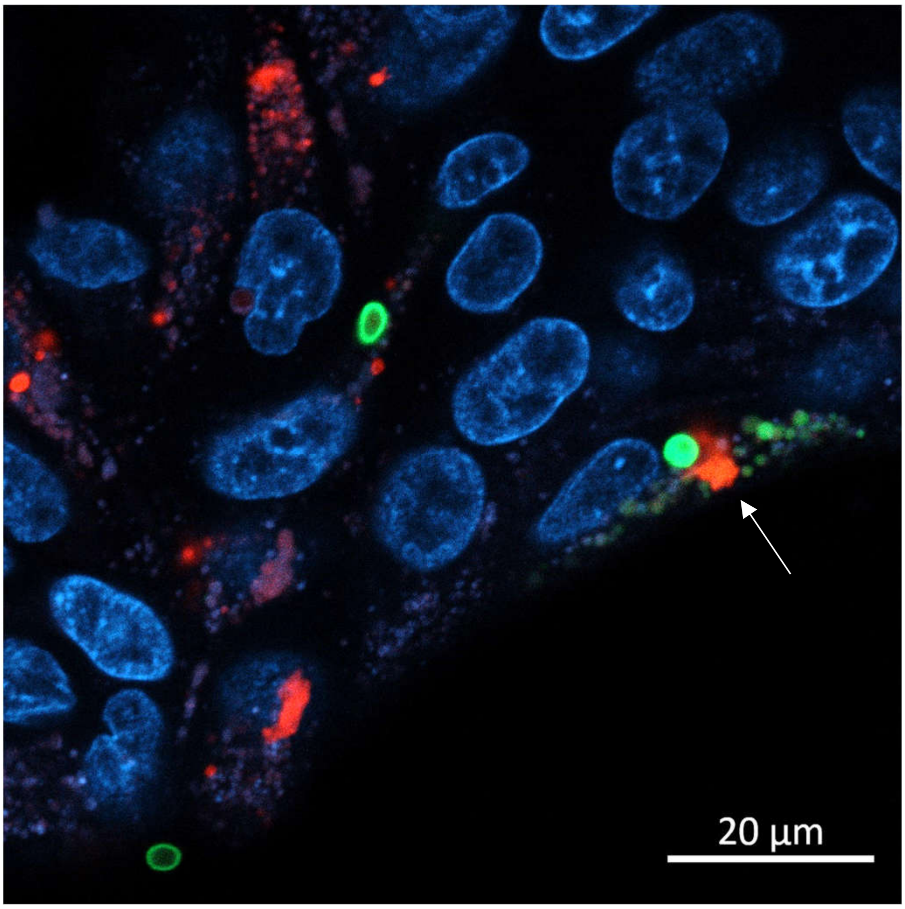

Supp Fig. S14: IECs can phagocytose both zymosan and *C. albicans*. Ileal (A) and colonic (B) organoids were fed overnight with pHrodo-red zymosan and Rhodamine-green-X labeled HK- *C. albicans* and stained with Hoechst 33342 prior to confocal live imaging. Arrows indicate cells that phagocytosed both zymosan and *C. albicans.* Original magnification x10 (A) x40 (B), scale bar 100 µm (A) and 20 µm (B).

Supp Fig S15

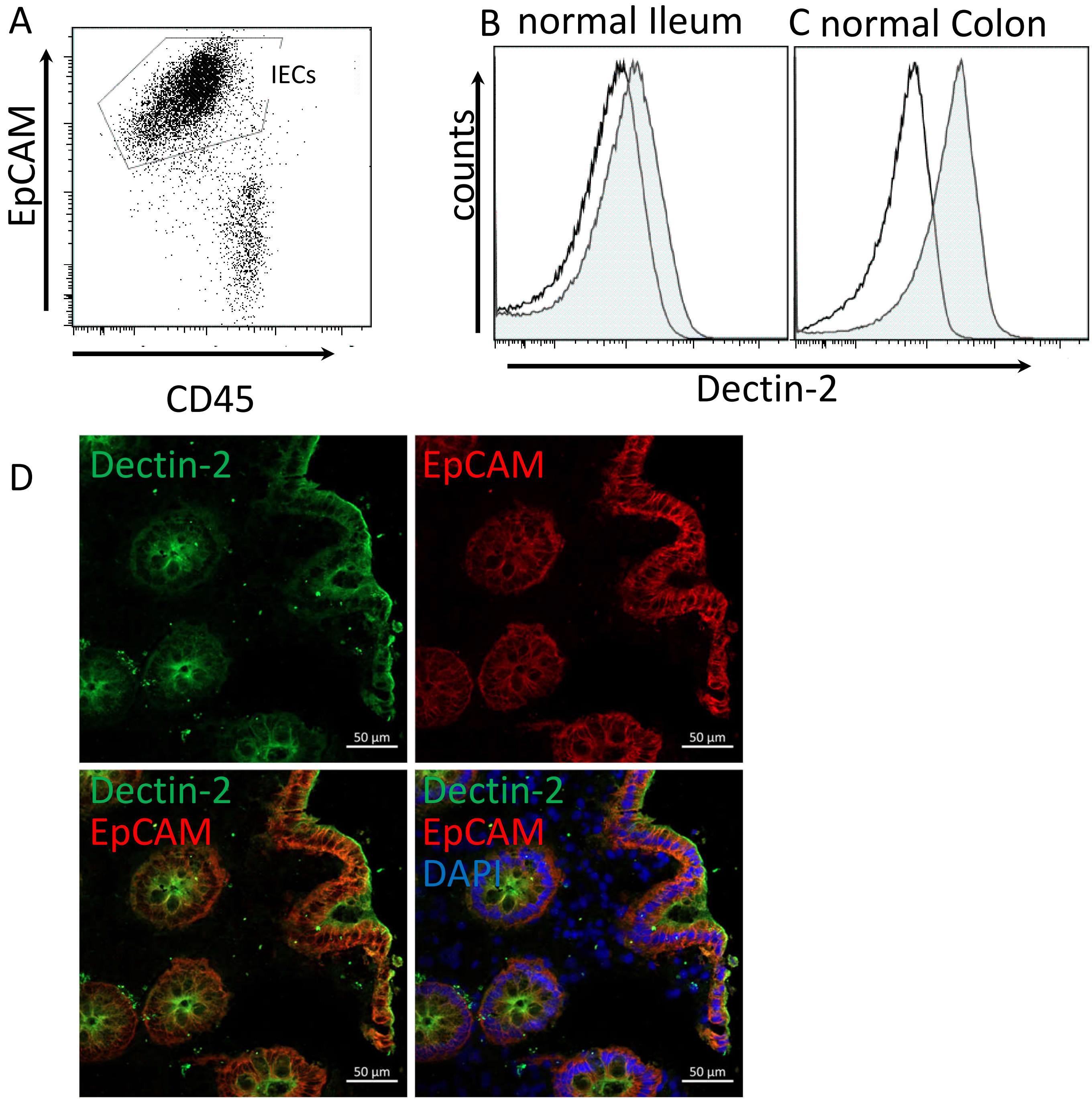

Supp Fig. S15: Dectin-2 is expressed on primary human IECs. (A) EpCAM-based flow cytometric gating on IECs. Freshly isolated human IEC-enriched preparation was stained with EpCAM and CD45 antibodies to identify IECs and leukocytes respectively. (B-C) Human IEC cell surface expression of Dectin-2. IECs generated from the (B) ileum and (C) colon of the same donor were stained as above, as well as with Dectin-2 Ab. Dectin-2 expression by intestinal epithelial cells (gated EpCAM+ population) was assessed by flow cytometry (filled gray histograms) and was compared with isotype-matched control Ab (open histograms). The data shown are representative of at least 3 individuals.

(D) Frozen sections of human colon surgical specimen were stained with Dectin-2 monoclonal antibody (green) and EpCAM polyclonal antibody (red) and counterstained with DAPI (blue). Original magnification x20, scale bar 50 µm. The images are representative of three individuals tested.

Supp Fig. S16

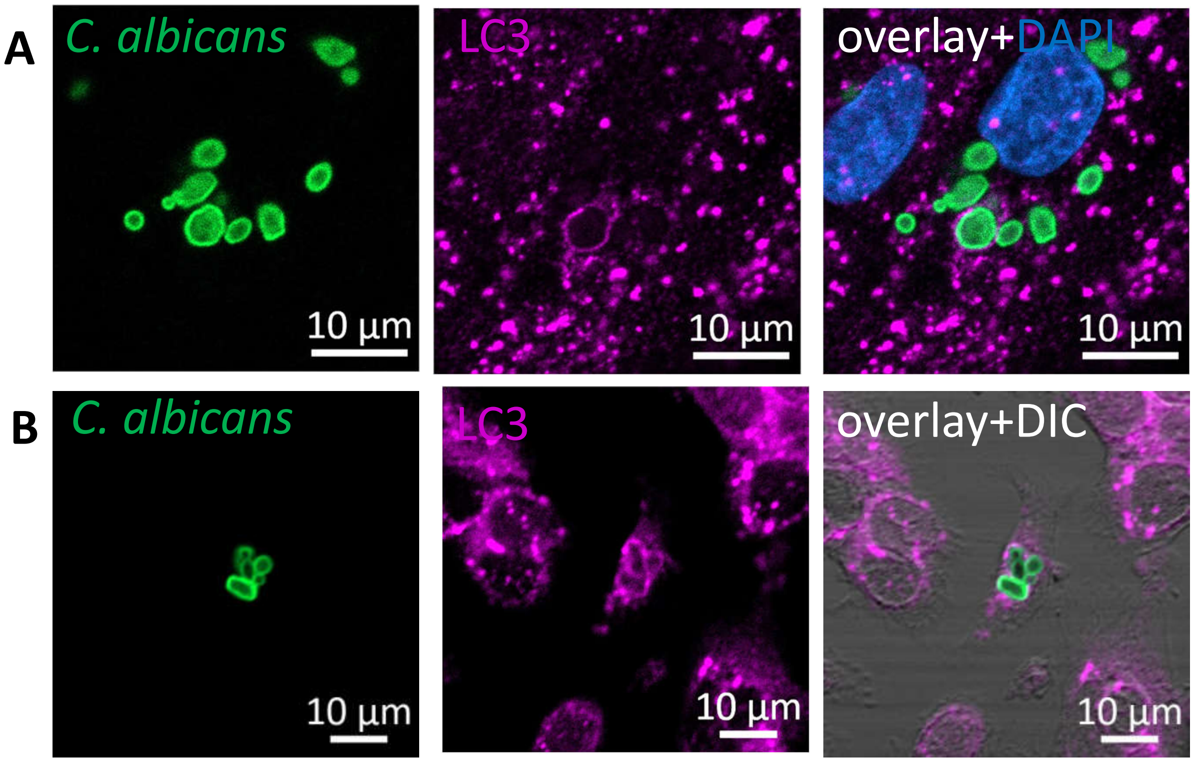

Supp Fig. S16: LC3 is recruited to phagocytosed particles.
Ileal organoids (A) and SW480 cells (B) were fed with Rhodamine-green-X HK- *C. albicans* and stained with LC3 (magenta) and DAPI (blue). Original magnification x63 (A) x20 (B), scale bar 10 µm.

Supp S17

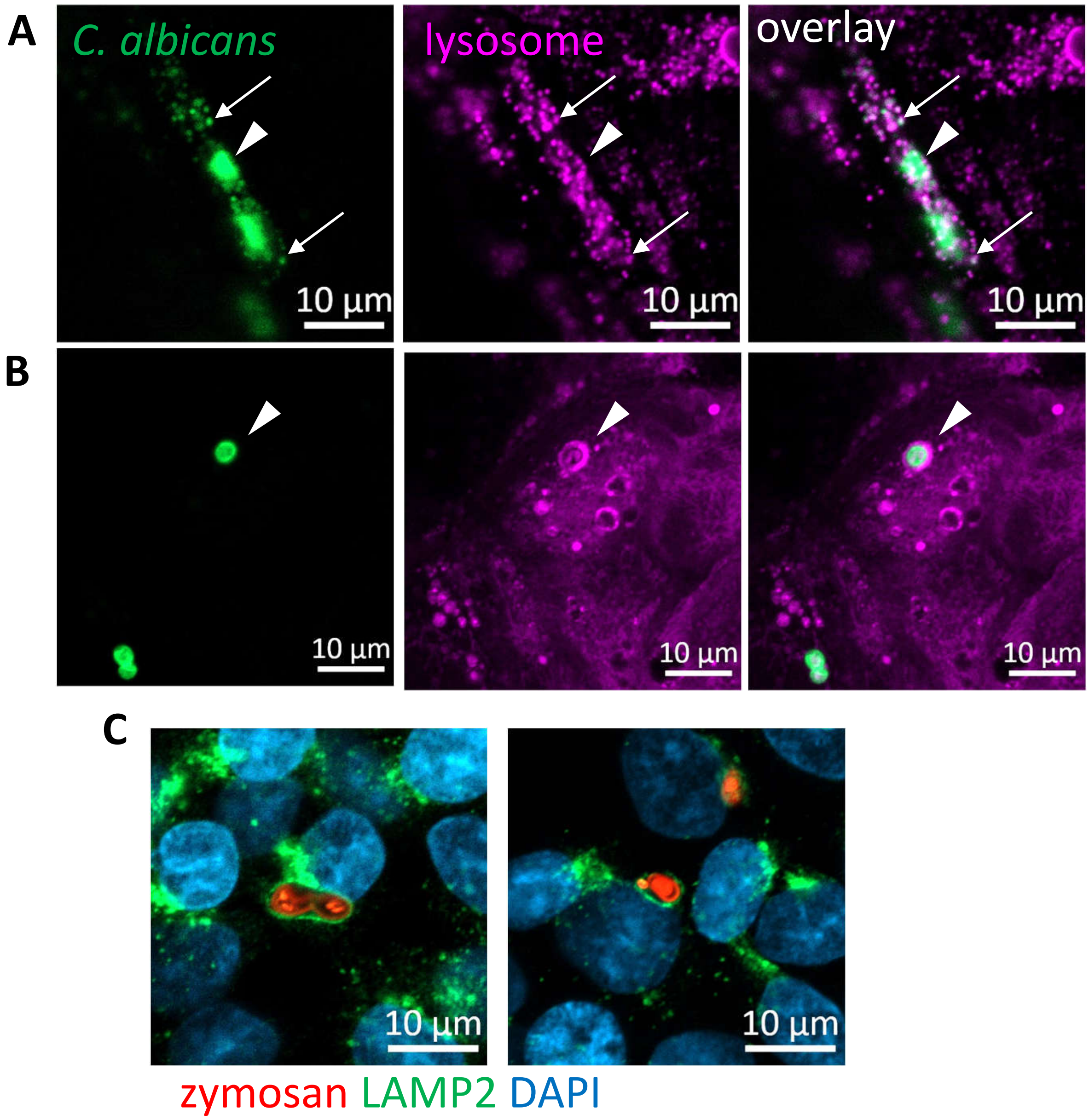

Supp Fig. S17: : Phagocytosed particles are directed to the lysosomes.
(A, B) Colonic organoids were fed with UV-inactivated *C. albicans* (green) and stained with lysosomal-NIR reagent (Magenta). Processed (A, arrow) and intact (A, B, arrowheads) particles are indicated, and co-localized with lysosomes. Original magnification x40 (A) x63 (B), scale bar 10 µm
C) HCT116 cells were fed with AF594-zymosan (red) and stained with LAMP2 antibody (green) and DAPI (blue). Original magnification x63, scale bar 10 µm
